# Understanding the genetics of root system architecture in pigeonpea [*Cajanus cajan* (L.) Millsp.]

**DOI:** 10.1007/s00122-025-05136-y

**Published:** 2026-01-23

**Authors:** Krishna B. Gaiwal, Shruthi H. Belliappa, Naresh Bomma, Satheesh Naik, N . Sandhyakishore, Sagar K. Rangari, Ashwini Kalyan, Shivaji P. Mehtre, Anilkumar Vemula, Rahul Bhosale, Manish K. Pandey, Harish Gandhi, Sean Mayes, Prakash I. Gangashetty

**Affiliations:** 1 International Crops Research Institute for the Semi-Arid Tropics (ICRISAT), Patancheru, Hyderabad, Telangana 502324 India; 2 Department of Genetics and Plant Breeding, Vasantrao Naik Marathwada Agricultural University, Parbhani, 431401 India; 3 Indian Institute of Agricultural Research, Pusa Campus (IARI), Samstipur, Bihar 848125 India; 4 Regional Agriculture Research Station, PJTSAU, Warangal, Telangana 506002 India; 5 School of Biosciences, University of Nottingham, Sutton Bonington Campus, Nottingham, LE12 5RD UK; 6 International Maize and Wheat Improvement Center (CIMMYT), ICRAF House, United Nations Avenue, Gigiri, Nairobi, 00621 Kenya

## Abstract

**Key message:**

Field-based phenotyping of root system architectural (RSA) traits in a diversity panel (PI-GAP) of pigeonpea was conducted across three diverse pigeonpea growing environments along with identification of genomic regions associated with these traits through GWAS analysis.

**Abstract:**

Root system architecture (RSA) plays a crucial role in plant stress tolerance mechanisms serving as the main route for water and nutrient acquisition, while also mediating plant-rhizosphere signalling. In the current study, an attempt was made to understand the genetic variability and genomic regions associated with RSA traits, as a relatively unexplored area of research in pigeonpea. The field-based “Shovelomics” approach was utilized to phenotype eight RSA traits: tap root length (TRL), lateral root length (LRL), number of lateral roots (NRL), stem diameter (SD), root diameter (RD), root angle from first and second lateral roots (RA1 and RA2) and root fresh weight (RFW) at physiological maturity. The pigeonpea international genome-wide association panel (PI-GAP) comprising of 185 genotypes from the reference set and 15 elite genotypes were used in the study. The combined ANOVA revealed significant genetic variance for all RSA traits except for RA2. Genome-wide association study was conducted using the *Axiom Cajanus 56 K SNP array*, leading to identification of 45 marker trait associations (MTAs) associated with RSA traits in pigeonpea. Multi-locus GWAS models detected six MTAs accounting for 4.84% to 18.73% of the phenotypic variation estimated (PVE) for TRL, 12 MTAs for LRL (4.73–13.92% PVE) and 11 MTAs for NLR (3.03–14.03% PVE value), respectively. Candidate gene analysis revealed genes associated with these traits, including *BAG* (Bcl-2-Associated athanogene) *family molecular chaperone regulator 6* (*CcLG01_17476096* and *CcLG01_17476721*), *root cap* (*CcLG04_5972718*) and *Protein MAINTENANCE OF MERISTEMS (MAIN)* (*CcLG06_8242342*). These genes were found to have key roles in growth and establishment of roots under stress-related conditions in model crops. Further validation of identified MTAs would provide an opportunity to develop trait-specific markers paving the way for marker-assisted breeding in pigeonpea. Based on RSA traits, pigeonpea genotypes were categorized into deep, spreading and dimorphic root system. These classifications facilitate the phenotypic selection of genotypes for breeding against drought, heat, waterlogging and salinity adaptation. Improved cultivars with an ideal root architecture designed for efficient resource uptake and high yield under diverse environments could help address food security challenges in semi-arid tropics.

**Supplementary Information:**

The online version contains supplementary material available at 10.1007/s00122-025-05136-y.

## Introduction

Root system architecture (RSA) refers to the spatial arrangement of various root components, including primary root, lateral root, basal root, their branching patterns, root density, root length, root angle and total root surface area (Lynch 2013; Kuijken et al. 2015). It plays a fundamental role in plant growth and adaptation by facilitating resource uptake, providing structural anchorage and serving as a storage unit for essential metabolites (de Dorlodot et al. 2007). Root traits have important role in the resource-limited environments, such as moisture limited (drought) and excessive (waterlogging) conditions. Deep and robust root systems enable plants to access water and nutrients from deeper soil layers, enhancing drought tolerance and nutrient uptake efficiency. Root architectural traits like increased lateral root proliferation and greater root hair length enable extensive soil exploration horizontally under abiotic condition like waterlogging and salinity (Vadez 2008; Amarapalli et al. 2022; Bakala et al. 2024). For decades, breeding efforts have predominantly focused on above-ground traits, often overlooking the root system despite its vital role in crop growth and yield. However, with the increasing challenges posed by climate uncertainty, attention has shifted towards modulating RSA to enhance crop productivity and resilience under resource-limited conditions (Lynch 2013; Kuijken et al. 2015) as well as to focus on interactions between the roots and the associated rhizosphere.

Being a major grain legume of the semi-arid tropics, pigeonpea [*Cajanus cajan* (L.) Millsp.] is a multi-utility crop integral to food, nutrition and livelihood security. The grain is majorly consumed as split grain (dal) in Asia and as a whole grain in Africa. It is rich in protein (20–22%) and micronutrients such as calcium (Ca), magnesium (Mg), iron (Fe) and zinc (Zn) (Kalyan et al. 2025; Susmitha et al. 2022). Apart from food, farmers value this crop for feed, fodder and fuel wood. The deeper tap roots along with dense lateral roots enhance soil fertility through symbiotic nitrogen fixation. They also play a role in eradication of soil erosion by tightly binding to roots and fixing atmospheric carbon. These attributes of the crop make it a crop of subsistence farming. It is cultivated in 5.7 million hectares (Mha) with an annual production of 5 million tonnes (Mt) with the productivity of 0.82 t ha^−1^ (FAOSTAT, 2024). The pigeonpea industry is valued at around USD 15.6 billion and is projected to grow to USD 31.8 billion by 2035 (FMI, 2025). The uncertain rains and increasing wetting and dry spells in agricultural lands pose challenges for high input crops like rice and maize providing opportunity for pigeonpea. As a crop of marginal soils, pigeonpea is barely explored in terms of its role in abiotic stress adaptations. In this regard, understanding the root traits alongside yield gains priority in research.

Pigeonpea undergoes epigeal germination and develops an embryonic root system comprising a primary tap root and lateral roots. The primary tap root is ortho-gravitropic, growing vertically into the soil, while lateral roots typically extend horizontally at specific gravitropic setpoint angles (Basu et al. 2007; Kuya and Sato 2011; Kirschner et al. 2024). Both tap root and lateral root mainly focuses on water and nutritional uptake from deeper layers as well as subsurface soil in crops like groundnut, common bean and cowpea (Li et al. 2025; Bochmann et al. 2025; Chen et al. 2024; Zhang et al. 2025). The exploration of diversity for RSA traits in pigeonpea gives prospect to understand the spatial distribution of the root system in pigeonpea along with identification of the donors to be used improvement of root system architecture with respect to abiotic stress.

Pigeonpea exhibits a wide range of maturity groups, spanning from extra early to late. The extra early having a maturity of < 120 days while, early and mid-early have a maturity of 121–150 and 151–165 days, respectively. The medium and late groups have a maturity of 166–180 and > 180 days, respectively. Each maturity groups are having the considerable advantages and limitations pertaining to abiotic stress. Extra early and mid-early maturity groups depict drought escape mechanism, while medium and late maturity group depicts sensitivity towards terminal moisture stress, a moisture limited conditions in soil. In India, most pigeonpea varieties grown in farmers’ fields are exposed to terminal moisture stress during the critical pod-filling stage which leads to yield losses up to 63% (Upadhyaya et al. 2012; Deshmukh et al. 2009). Hence, in-depth understanding of RSA would help in improvement of dimorphic root system which indirectly contribute to drought tolerance (Burridge et al. 2020; Lynch 2019; Lynch et al. 2024).

Despite the significance of root traits in crop adaptation, root phenotyping remains a major challenge due to the complexity and opacity of soil environments. Greenhouse-based phenotyping methods provide controlled conditions for high-throughput screening (York et al. 2013). However, greenhouse trials typically rely on pots, which limit soil volume and restrict root growth and development (Poorter et al. 2012). Field-based phenotyping is crucial for capturing genotype × environment (G × E) interactions. In this regard, shovelomics emerges as a low-cost field phenotyping technique enabling breeders to assess root traits in natural soil conditions. This method involves excavating root systems, washing them and analysing their architecture using phenotyping boards or imaging (Trachsel et al. 2011; Burridge et al. 2016). Furthermore, the absence of genetic studies on RSA traits in pigeonpea limits RSA-based breeding. Hence, incorporating RSA trait into breeding programmes can enhance targeted genetic improvement for abiotic stress adaptation. Integrating GWAS with detailed phenotyping of RSA traits offers new opportunities to identify candidate genes and genomic regions underlying root development, paving the way for marker-assisted selection and the genetic improvement of the crop (Kim et al. 2023; Donde et al. 2023; Jiang et al. 2025).

Given the crucial role of RSA in conferring abiotic stress resilience, study was initiated to investigate the genetic potential of RSA traits in pigeonpea. While previous research using shovelomics has highlighted RSA traits in other crops like cowpea, soybean and common bean, a comprehensive understanding in pigeonpea remained unexplored. Therefore, this investigation systematically assessed the genetic variability and genome-wide association studies (GWAS) for RSA traits within the Pigeonpea International Genome Wide Association Panel (PI-GAP). The aim was to identify promising trait donors and associated markers which will subsequently be incorporated into breeding programmes.

## Materials and methods

### Plant material

The current study material consisted of 185 reference set accessions and 15 elite genotypes from the Pigeonpea International Genome Wide Association Panel (PI-GAP) sharing the origin across 25 countries worldwide (Table S1; Fig. S1). The seeds were collected from Rajendra Singh Paroda Genebank, ICRISAT, and single plant selection was carried out for two seasons as a purification process before deriving a uniform accession. This panel was selected for its extensive genetic diversity, capturing variability across multiple morpho-physiological and yield-related traits.

### Field experiment

The experimental trial was laid out in an alpha lattice design for 200 genotypes with two replications, using a block size of 10. Each genotype was planted in two rows of 3.0-m length, with an inter row spacing of 75 cm and plant to plant spacing of 15 cm, providing a plot size of 4.5 m^2^ (0.75 × 3 × 2). The field trials were conducted during rainy-2023 across three distinct environments: International Crops Research Institute for the Semi-Arid Tropics (ICRISAT), Patancheru, India (17.51°N latitude, 78.27°E longitude, 545.00 m above mean sea level), Indian Institute of Pulses Research (IIPR), Kanpur, India (26.27°N latitude, 80.14°E longitude and 125.00 m above mean sea level) and Regional Agricultural Research Station (RARS), Warangal, India (15.50°N latitude, 79.28°E longitude, 268.50 m above mean sea level), whereas sowing was done on 16 June 2023 at ICRISAT-Patancheru and IIPR-Kanpur and on 24 June 2023 at RARS-Warangal. The environmental conditions varied across environments. At ICRISAT-Patancheru, the average maximum and minimum temperatures were 31.8 and 21.5 °C, respectively, with 923.18 mm total rainfall and 86.33% relative humidity (RH), while RARS-Warangal recorded the temperatures 32.5 °C (max), 21.55 °C (min), 954.80 mm rainfall and 85.97% RH. The third environment, IIPR-Kanpur, recorded the temperatures 30.74 °C (max) and 19.21 °C (min), with 859.10 mm rainfall and 84.14% of RH in Table S2. The soil type too varied at the experimental sites with Vertisols type at ICRISAT-Patancheru, Alfisols at RARS-Warangal and Inceptisols at IIPR-Kanpur being a major soil type. The details of the soil profiles of three experimental sites are provided in Table S3.

### Shovelomics: a root phenotyping protocol

Shovelomics is a field-based phenotyping method that involves manually excavating plants using standard shovels (Trachsel et al. 2011; Burridge et al*.* 2016). This method makes it possible to thoroughly characterise the root morphology and architecture under typical growing circumstances, offering important insights into root traits associated with plant performance and stress tolerance. As root architecture studies in pigeonpea are a first of its kind, standardizing the Shovelomics protocol represented a pioneering step in this research. The protocol included the following steps.

### Root excavation

Three plants were randomly selected for root phenotyping. The physiological maturity stage was identified as the optimal time for phenotyping, as root growth and establishment could be considered evident at this stage. To facilitate root excavation, the experimental field was irrigated to field capacity for consecutive days. On the day of excavation, running irrigation was applied to soften the soil further, allowing for easier manual excavation with minimal root damage. Roots were excavated using a standard spade, which enabled careful uprooting of the primary tap root along with long, horizontally spread lateral roots and intact fine tertiary roots. This ensured a complete and representative root sample for phenotypic assessment. Each selected root crown was carefully tagged and labelled for accurate identification prior root washing.

### Root cleaning and washing

A standardized cleaning protocol was applied for excavated roots before trait measurements to maintain precision. Accordingly, the following steps were followed.

1. Removal of soil particles: Briefly shaking root crowns to remove excess soil.
2. Detergent soaking: Submerging roots in a 0.5% of detergent solution for 10–15 min to detach remaining soil particles.
3. Cleaning: Rinsing of root vigorously under low water pressure to eliminate any residual soil particles adhering to the roots.
4. Shade drying: Thoroughly cleaned roots were dried for 10 min under shade to remove excess water before measurements.

### Phenotyping and trait measurement

**Fig. 1:**
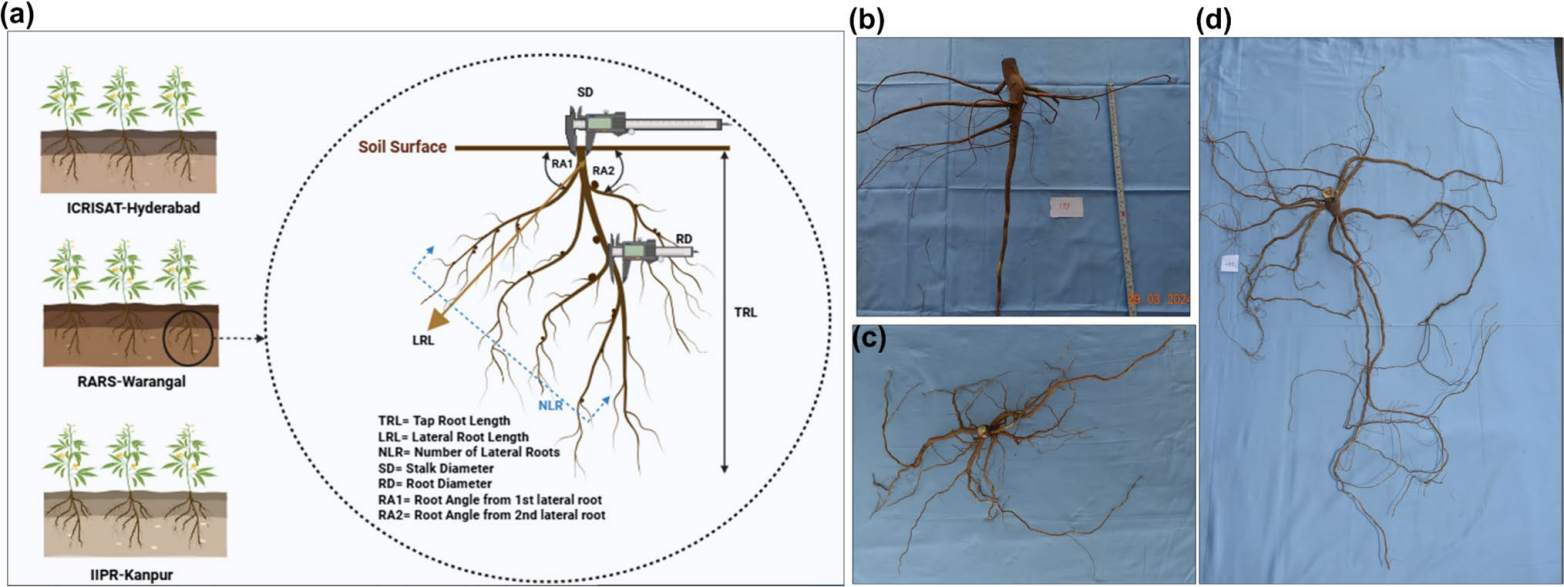
Root System Architecture (RSA) of pigeonpea: **a** RSA traits evaluated across three diverse environments: ICRISAT-Patancheru, IIPR-Kanpur and RARS-Warangal; **b** Genotype exhibiting a deep root system; **c** Genotype with a spreading root system **d** Genotype showing a dimorphic root system

**Fig. 2:**
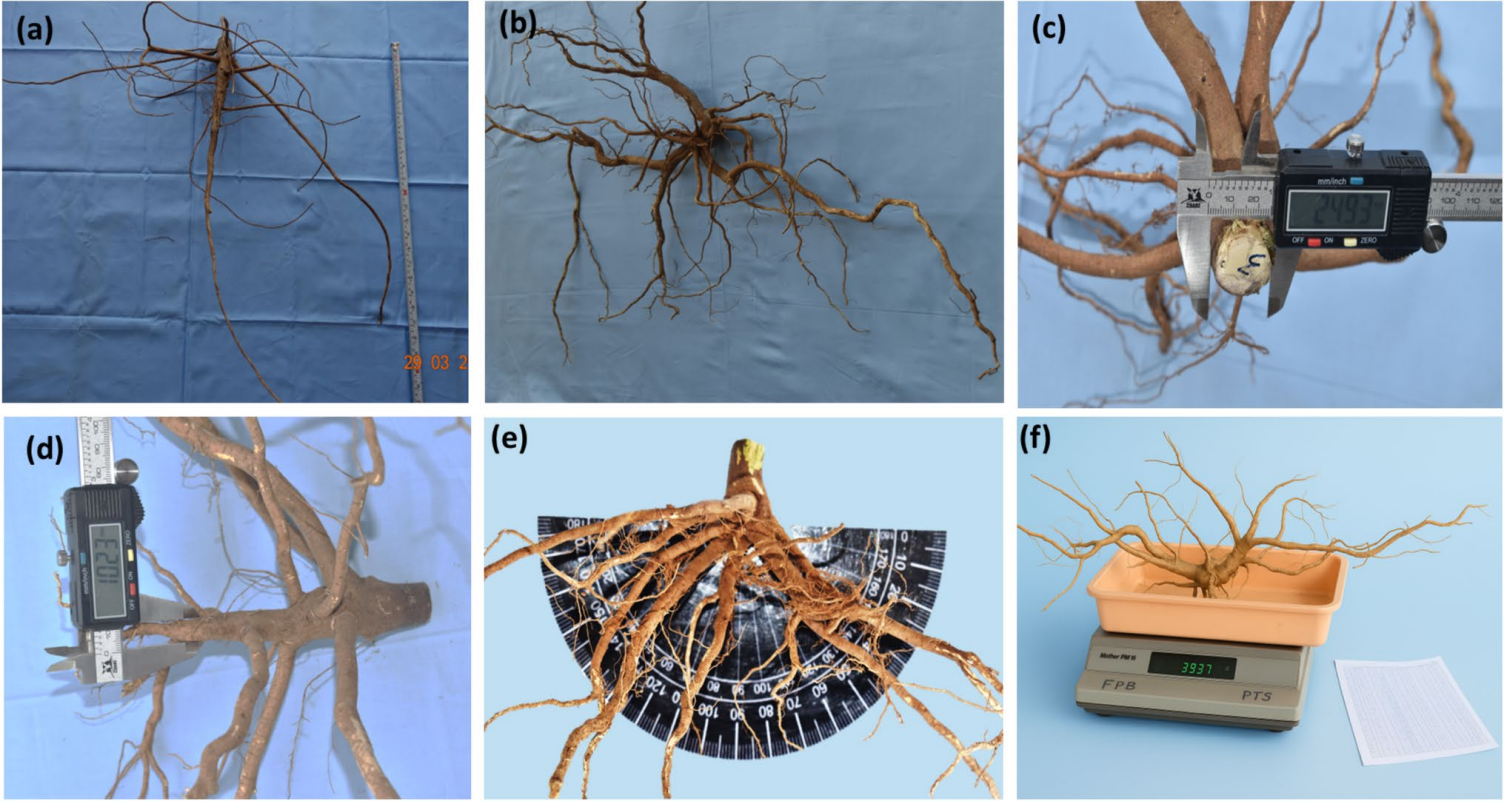
Shovelomics phenotyping of root system architectural traits in pigeonpea including: **a** tap root length (cm), measured using a meter tape; **b** lateral root length (cm) and number of lateral roots, also measured using a meter tape and manual counting, respectively; **c** stem diameter at the soil level (cm), measured with a vernier calliper; **d** tap root diameter 10 cm below the stem (cm), measured with a vernier calliper; **e** root angle from the first and second lateral roots (°), measured using a protractor; and **f** root fresh weight measured with a weighing balance

Cleaned root samples were arranged on a phenotyping board specifically designed for precise root trait measurement. The phenotyping measuring board is a simple wooden frame of 1 m height; 2 m length; and 1 m width without a grid or section marking. The roots were horizontally placed on the board, and the trait measurements were taken for RSA traits with the help of measuring tape, vernier calliper, 180° protractor and precision weighing balance. Roots were measured sequentially for all traits, and data were recorded. The following eight RSA traits were measured using standardized protocols as shown in Figs. 1 and 2.

1. Tap root length (TRL): Distance from the base of the stem to the tip of the tap root was measured in centimetres using a measuring tape.
2. Lateral root length (LRL): Length of lateral roots emerging from the tap root was measured in centimetres using a measuring tape.
3. Number of lateral roots (NLR): A number of lateral roots branching from the tap root were manually counted.
4. Stem diameter (SD): Stem diameter was measured at the soil surface using an electronic digital vernier calliper (Model 5HA 1890, Omni-Tech Electronic Co., Limited, Hong Kong, China). The vernier calliper was adjusted at the cut crown of the stem, and digital reading was recorded in centimetres
5. Tap root diameter (RD): A tap root diameter was measured at 10 cm below the soil surface by adjusting the vernier calliper around root to record the reading.
6. Root angle 1 (RA1): Roots were laid on the 180° protractor placed on the phenotyping board; root stem was then kept at the centre point of protractor such that tap root is parallel to 90° line. A root angle between first lateral root and the reference line was recorded manually.
7. Root angle from second lateral root (RA2): RA2 was measured similarly to RA1 for the second lateral root.
8. Root weight (RFW): Roots were placed on the precision weighing balance (Mettler PM16) and the weight was recorded in grams.

### Categorizing root systems based on RSA traits

Roots were further classified into three distinct types, based on to their architectural characteristics. This classification was derived from measurable traits such as tap root length, lateral root length, the number of lateral roots, root diameter, stem diameter and root angles (Burridge et al. 2020). Accordingly, roots were classified into:

### Deep root system

Roots growing predominantly downward with deeper tap root length and shorter lateral roots were classified as deeper root system. In such system, a number of lateral roots were minimal, whereas root diameter and stem diameter were found to be higher with steeper root angle (Fig. 1b).

### Spreading root system

Roots spreading horizontally near the soil surface with longer and numerous lateral roots were termed as spreading root system. They had higher stem diameter and a shallow root angle (Fig. 1c).

### Dimorphic root system

Roots growing both deeper and horizontal to the soil surface layer were classified as dimorphic root system. Such roots had lengthier tap roots, longer lateral roots, moderate root as well as stem diameter, with lesser root angle (Fig. 1d).

The observations generated from the study were further utilized for assessing genetic variability. As there were sequenced data for the genotypes under the study, marker trait associations (MTAs) linked with these root traits were identified.

### Genotyping using Axiom Cajanus SNP array with 56 K SNP data

The Axiom *Cajanus* 56 K SNPs array genotyping platform was developed from the sequence of 103 pigeonpea lines. A total of 56,512 unique and informative sequence variants were included in this genotyping array (Saxena et al. 2018). The genotyping for 185 lines used in the present study was done using this Axiom *Cajanus* 56 K SNPs array. High-quality DNA was isolated from fresh leaves of each genotype using the NucleoSpin Plant II kit (Macherey–Nagel). The quality of the extracted DNA was checked on 0.8% agarose gel electrophoresis, and DNA quantification was performed using a Qubit 2.0 Fluorometer (Life Technologies Thermo Fisher Scientific Corp.). Target probes were prepared using high-quality DNA (20 μL of 10 ng μL^−1^) from each line following “Affymetrix Axiom 2.0” procedure. Further, DNA samples were amplified, fragmented and hybridized on the chip followed by single-base extension through DNA ligation and signal amplification. *Affymetrix GeneTitan* was used for staining and scanning samples. Alternate and reference alleles for each SNP markers were detected using Axiom Analysis Suite version 1.0. (http://media.affymetrix.com /support/downloads/manuals/Axiom analysis_suite_user_guide.pdf). Allele assignment was performed based on the pigeonpea reference genome ICPL 87119 (v1.0). The suggested genotyping workflow (Best Practices Workflow) was used for the genotyping of imported CEL files.

## Statistical analysis

### Variance and genetic parameters

A combined analysis of variance (ANOVA) was used to analyse the main and interaction effects of environment and genotypes, with environment, replication, the nested effect of block with replication and genotype treated as random. Individual variances of environments were estimated and modelled to error distribution using the residual maximum likelihood (REML) algorithm in SAS Mixed (SAS v9.4, SAS Institute Inc. 2023). BLUPs (Best Linear Unbiased Predictors) were computed for genotypes (*G*), environment and environment × genotypes using a combined analysis of variance.

The estimates of repeatability for each RSA trait across environment were categorized into low (< 0.40), medium (0.41–0.69) and high (> 0.70) (Falconer and Mackay 1996). Correlation coefficients among RSA traits were computed using the native R function *cor ()* and visualized with the *corrplot* package of RStudio (Wei and Simko 2017). The Multi-Trait Genotype–Ideotype Distance Index (MGIDI) was used to select genotypes with ideal performance across multiple RSA traits (Olivoto and Nardino 2020).

### Genetic diversity estimation, PCA (Q matrix) and kinship relatedness (K matrix)

The diversity of the reference set genotypes was estimated using the Tassel software based on the nucleotide diversity (Bradbury et al. 2007). The Unweighted Pair Group Method with Arithmetic Mean (UPGMA) statistics were employed to construct a dendrogram for the 185 genotypes. The resulting “NWK” extension file was subsequently used in the “iTOL” webtool (https://itol.embl.de/) to improve the representation of the tree. Principal component analysis (PCA) was conducted using the GAPIT package of RStudio (Lipka et al. 2012). To identify sub-populations within the 185 genotypes additionally, the genetic relationships among the selected genotypes were evaluated using kinship matrix (*K*), which helps correct false positives and is likely to provide the true marker trait associations.

### Genome-wide association mapping

In the Axiom *Cajanus* 56 K SNPs array containing a total of 56,512 markers (SNPs and Indels), filtration was done with minor allele frequency (MAF) ≥ 0.05 and maximum heterozygous proportion 25%. Following filtration, the trait-specific SNPs were identified through Multi-locus Random-SNP-Effect Mixed Linear Model (mrMLM) and The Genomic Association and Prediction Integrated Tool (GAPIT) version 3 package of RStudio. Furthermore, within mrMLM framework, six different association models were implemented including (1) Multi-Locus Random-SNP-Effect Mixed Linear Model (mrMLM), (2) Fast Multi-Locus Random-SNP-Effect Mixed Linear Model (FASTmrMLM), (3) Fast Multi-Locus Mixed-Model Association (FASTmrEMMA), (4) Polygenic-background-control-based Least Angle Regression and Empirical Bayes (pLARmEB), (5) Polygenic-background-control-based Kruskal–Wallis test and Empirical Bayes (pKWmEB), (6) Iterative Sure Independence Screening Extended Empirical Bayes LASSO (ISIS EM-BLASSO) (Zhang et al. 2020a, b). For the mrMLM models, SNPs with a LOD score greater than 3 were considered significantly associated with the trait. In the GAPIT model, genome-wide threshold was determined using the Bonferroni correction, calculated as 0.05/37865 = 1.32048E-06, corresponding to − log10 (*p* value) = 5.87. SNPs that met the significance threshold were defined as significant (Dudbridge and Gusnanto 2008).

### Allelic distribution of the identified significant MTAs

The allelic distribution of all the markers was checked in the 185 genotypes. The distribution of favourable and unfavourable alleles in the extreme genotypes was compared, and significant markers having associations with RSA were selected.

### Identification of candidate gene

Systematic process to identify candidate genes for RSA-related traits was used. Candidate genes were fetched from the 58 kb upstream and downstream region of the identified MTAs. The genes filtered based on their predicted functions, and those likely related to the trait of interest were retained as candidate genes. The function of these genes was identified using KEGG annotation file of pigeonpea draft genome version 1 (ICPL 87119) (Varshney et al. 2012). The function of unknown genes was identified in Interproscan (https://www.ebi.ac.uk/interpro/search/sequence/) webtool using the amino acid sequence of the genes.

## Results

### Estimates of variance components for genotypes, environment and their interactions

From the combined analysis of variance (Table 1), all traits except RA2 showed significant genetic variation and genotype × environment interaction (*p* < 0.05). The main effect of environment is substantial for all RSA traits, indicating that environment variances were high.

**Table 1:** Estimation of variance components for root architectural traits among 200 pigeonpea genotypes evaluated across ICRISAT-Patancheru; RARS-Warangal and IIPR-Kanpur

Traits	Components	Genotypes	Environment	Genotypes x environment	Environment x replication	Environment x replication x block	ICRISAT, Patancheru	RARS, Warangal	IIPR, Kanpur
TRL	Variance	2.75	37.19	6.28	0.09	2.59	35.21	9.59	50.39
	SE	1.15	37.42	1.62	0.27	0.81	3.47	1.06	4.7
	Prob. chi-square	0.0092	0.0006	< 0.0001	0.6714	< 0.0001	–	–	–
LRL	Variance	19.45	5.72	19.46	0	5.76	64.43	61.89	81.78
	SE	4.39	6.18	4.97	–	2.13	7.12	6.44	8.17
	Prob. chi-square	< 0.0001	0.0293	< 0.0001	–	0.0006	–	–	–
NLR	Variance	0.76	1.81	0.57	0.07	0.29	4.54	1.83	4.61
	SE	0.18	1.87	0.2	0.08	0.1	0.44	0.2	0.44
	Prob. chi-square	< 0.0001	0.0107	0.0024	0.061	0.0001	–	–	–
SD	Variance	0.03	0.26	0.01	0	0.02	0.07	0.06	0.24
	SE	0.01	0.26	0.01	–	0	0.01	0.01	0.02
	Prob. chi-square	< 0.0001	0.0003	0.0226	–	< 0.0001	–	–	–
RD	Variance	0.02	0.05	0.02	0	0.01	0.03	0.03	0.1
	SE	0	0.05	0	–	0	0	0	0.01
	Prob. chi-square	< 0.0001	0.0003	< 0.0001	–	< 0.0001	–	–	–
RA1	Variance	2.24	2.28	0	0	0.81	33.85	26.73	43.91
	SE	0.87	2.4	–	–	0.6	2.81	2.16	3.41
	Prob. chi-square	0.0067	0.0085	–	–	0.1294			
RA2	Variance	1.78	6.84	1.37	0	1.35	54.1	26.58	59.25
	SE	1.16	7.01	2.02	–	0.79	4.72	2.63	4.83
	Prob. chi-square	0.1062	0.0057	0.4884	–	0.0463	–	–	–
RFW	Variance	189.3	271.2	285.7	1.5	35.3	115.2	106.9	447.3
	SE	38	275.6	34.7	3.6	10.1	13.9	13.3	59.4
	Prob. chi-square	< 0.0001	0.0015	< 0.0001	0.5541	< 0.0001	–	–	–

TRL: Tap root length; LRL: Lateral root length; NLR: Number of lateral roots; SD: Stem diameter; RD: Root diameter; RA1; Root angle from 1st lateral root; and RA2: Root angle from 2nd lateral root; RFW: Root fresh weight; SE: Standard error; and Prob. Chi-square: Probability of chi-square

### The mean performance of the genotypes for RSA traits

The mean values and distribution of variation across genotypes for multiple RSA traits are presented in Fig. S2. The mean performance of the genotypes in the study varied for trait TRL ranging from 13.17 to 37.75 cm, with a mean of 24.87 cm. The LRL varied from 26.75 to 63.08 cm, with trial mean of 43.03 cm. Similarly, the NLR varied from 5.58 to 13.39 with trial mean of 9.57. The SD varied from 0.98 to 2.55 cm, with a mean of 1.95 cm. The RD ranged from 0.52 to 1.53 cm, with a mean of 0.98 cm. The RA1 ranged from 10.00° to 25.83°, with a mean of 16.55°. Similarly, the RA2 ranged from 12.5° to 31.39° with a mean of 19.81°. The RFW ranged from 9.83 to 131.50 g, with an average of 52.91 g (Table 2).

**Table 2:** Mean, range and genetic variability parameters of root architectural traits in pigeonpea genotypes

Traits	Mean	Above average genotypes	Range		CV %	GCV	PCV	Repeatability (%)	GAM (%)
			Min	Max					
TRL (cm)	24.87	99	13.17	37.75	22.38	6.59	12.65	27.00	7.06
LRL (cm)	43.03	102	26.75	63.08	19.21	10.17	14.13	52.00	15.07
NLR	9.57	106	5.58	13.39	19.77	8.99	12.89	49.00	12.88
SD (cm)	1.95	106	0.98	2.55	17.82	8.39	11.55	53.00	12.54
RD (cm)	0.99	94	0.52	1.53	23.34	13.36	18.02	55.00	20.38
RA1 (°)	16.56	95	10.00	25.83	35.35	8.96	16.99	28.00	9.73
RA2 (°)	19.81	99	12.50	31.39	34.11	6.66	15.80	18.00	5.78
RFW (gm)	52.91	92	9.83	131.50	28.38	26.14	34.07	59.00	41.33

TRL: Tap root length; LRL: Lateral root length; NLR: Number of lateral roots; SD: Stem Diameter; RD: Root diameter; RA1; root angle from 1st lateral root; RA: Root angle from 2nd lateral root; RFW: Root fresh weight; GCV: Genotypic coefficient of variance; PCV: Phenotypic coefficient of variance, GA: Genetic advance and GAM: Genetic advance as per cent of mean

### Genetic variability and character association among RSA traits

**Fig. 3:**
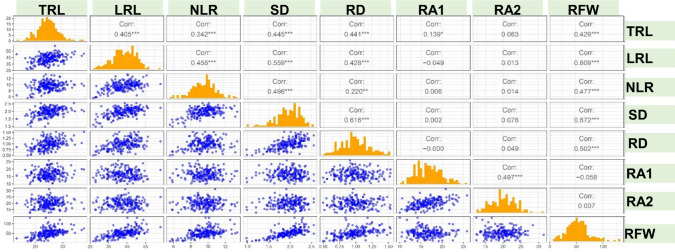
Graphical illustration of Pearson’s correlation among root system architectural traits of pigeonpea, including: tap root length (TRL, cm); lateral root length (LRL, cm), number of lateral roots (NLR); stem diameter at the soil level (SD, cm); root diameter 10 cm below the stem (RD, cm); root angle from the first and second lateral roots (RA1, RA2, °); and root fresh weight (RFW, g)

The genetic variability analysis of root traits in pigeonpea revealed considerable variation, as indicated by the estimated genotypic coefficient of variation (GCV), phenotypic coefficient of variation (PCV), repeatability and genetic advance as a percentage of the mean (GAM). The GCV varied from 6.59 (TRL) to 26.14 (RFW). Similarly, the PCV ranged from 11.55 (SD) to 34.07 (RFW). The repeatability and GAM for RSA traits ranged from 18% (RA2) to 59% (RFW) and 5.78 (RA2) to 41.33 (RFW), respectively. The traits RFW and RD have high GAM (> 20%) and repeatability, which indicated additive gene action and selection is likely to be more successful for the respective traits. The moderate-to-high GAM (10% to 20%) and repeatability of the traits LRL, NLR and SD indicated that selection may improve these traits. The low GAM (< 10%) and repeatability observed for RA1, RA2 and TRL indicate that these traits are strongly influenced by the environment, making phenotypic selection less effective. The coefficient of variation CV (%) for each of the 8 traits ranged from 17.82% (SD) to 35.35% (RA1) (Table 2). A significant positive correlation was observed between TRL and LRL (*r* = *0.405, P* < *0.001*) and between SD and RD (*r* = *0.616, P* < *0.001*). The trait RFW showed significant a positive correlation with TRL (*r* = *0.429, P* < *0.001*), LRL (*r* = *0.609, P* < *0.001*), NLR (*r* = *0.477, P* < *0.001*), SD (*r* = *0.672, P* < *0.001*) and RD (*r* = *0.502, P* < *0.001*) (Fig. 3).

Desirable genotypes for each RSA trait were identified based on the combined values of mean and standard error. Genotypes ICP 4715 and ICP 8211 were found for TRL, while ICP 12105 and ICPL 20202 were found superior for LRL. For the number of NLR, ICP 10508 and ICPL 20201 were the best performers. Genotypes ICP 14444 and ICP 12515 showed superior performance for SD. In terms of RD, ICP 4715 and ICP 9750 were identified as promising, whereas ICP 7337 and ICP 11969 performed well for RFW. Genotypes ICP 11230 (MGIDI = 2.824), ICP 12515 (MGIDI = 2.959), ICP 11833 (MGIDI = 3.161) and ICP 10559 (MGIDI = 3.166) were selected as an ideal genotypes based on the Multi-Trait Genotype–Ideotype Distance Index (MGIDI) for root system architectural traits (Table S1).

### Genetic diversity, SNP distribution, linkage disequilibrium

**Fig. 4:**
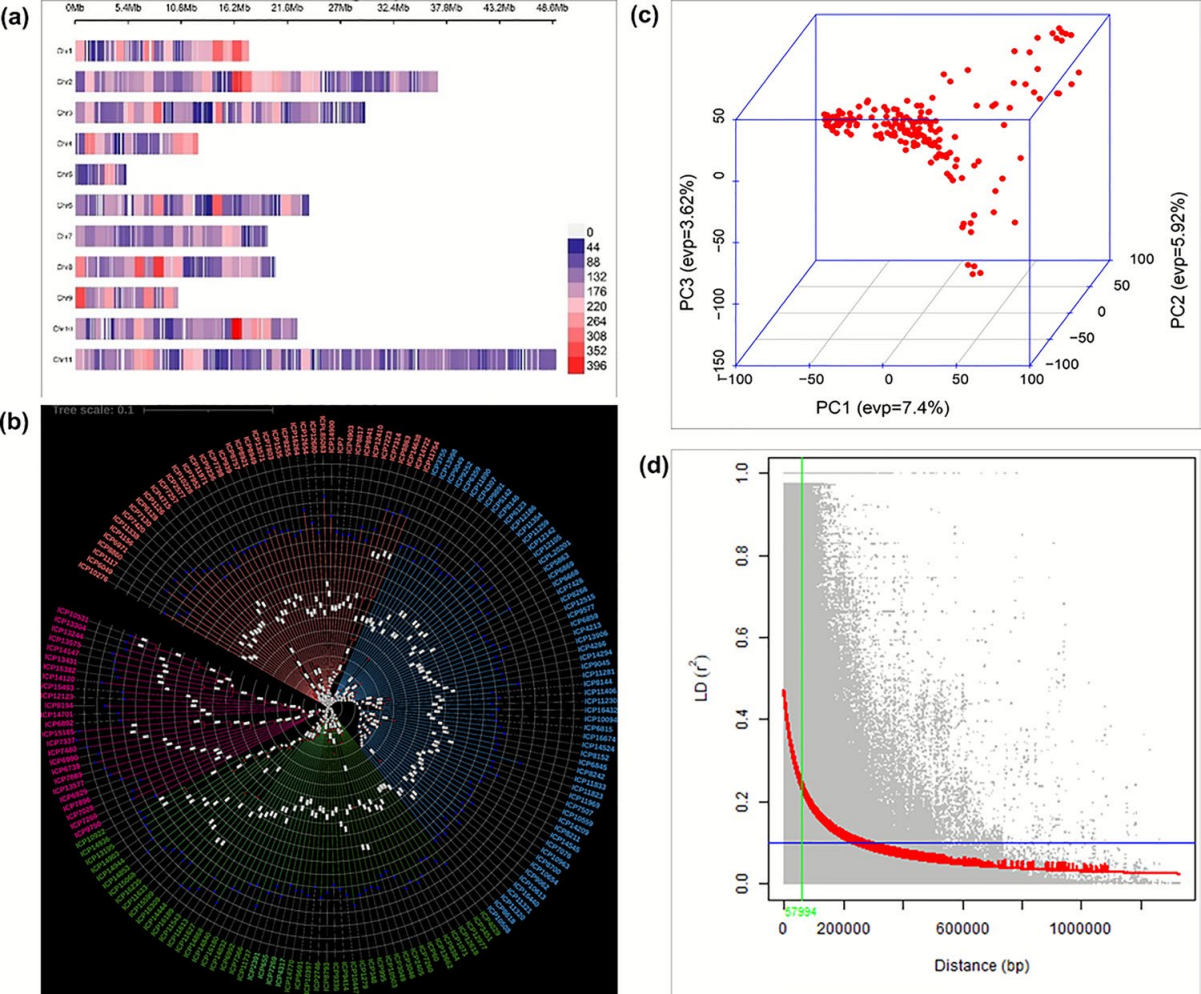
Genetic analysis of the PI-GAP panel of pigeonpea, showing **a** distribution of SNPs across the 11 chromosomes, **b** genotypic diversity among reference set genotypes, **c** principal component analysis (PCA) of reference set genotypes and **d** genome-wide average linkage disequilibrium (LD) decay over physical distance

The *Axiom Cajanus 56 K SNPs array* contains a total of 56,512 markers (SNPs) after filtrations of 37,865 biallelic SNPs remained for 185-genotype panel. The filtered SNPs were distributed across all 11 pigeonpea chromosomes (CcLG01–CcLG11), with the highest number observed on CcLG02 (5,942) and CcLG11 (5,955), while the lowest was on CcLG05 (667). This genome-wide distribution provides balanced coverage, with an average SNP density ranging from ~ 1 per 3.4 kb to ~ 1 per 7.9 kb, facilitating a robust genotypic analysis (Fig. 4a) and indicating that the marker set was well suited for diversity analysis and GWAS for RSA in pigeonpea.

Using the 37,865 polymorphic SNPs, genetic distance mapping was undertaken on 185 genotypes from the PI-GAP which classified the entire panel into four clusters. Cluster II was the largest followed by clusters III, I and IV with 62, 54, 43 and 26 genotypes, respectively (Fig. 4b). The PCA results showed 185 pigeonpea genotypes divided into four different sub-groups with overlapping areas. PC1, PC2 and PC3 accounted for 8.28, 1.94 and 1.62%, respectively (Fig. 4c.). The linkage disequilibrium (LD) of each pair of SNPs across the genome was evaluated by a squared Pearson correlation coefficient (*R*^2^). The analysis produced half LD decay value of 57,994 bp (57.99 kb) (Fig. 4d). Thus, a 58 kb upstream and downstream region from significant MTAs was considered for identifying the trait-associated gene.

### Genome-wide association studies for root system architecture (RSA)

**Fig. 5:**
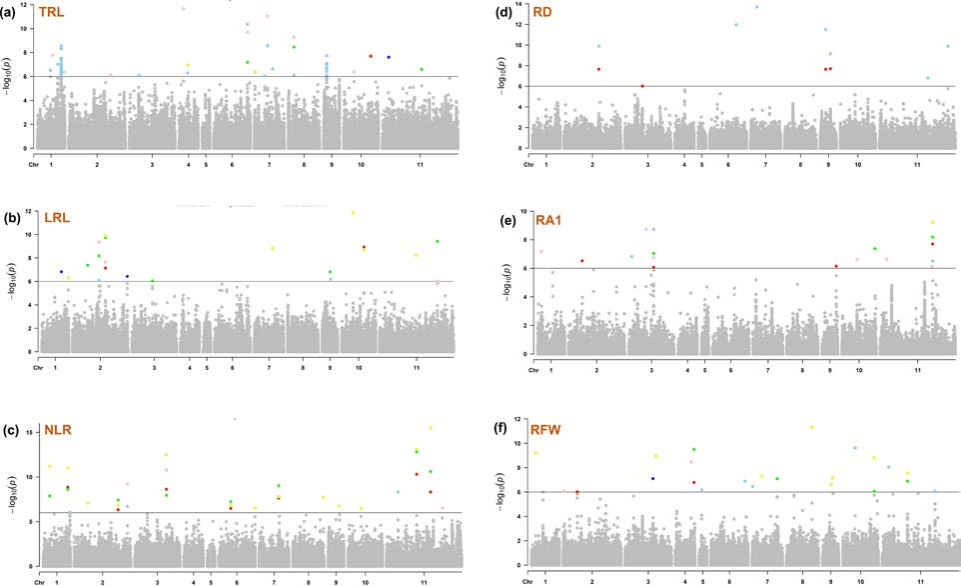
Manhattan plots of multi-locus genome-wide association studies of root system architecturral traits using a 185 genotypes from Pigeonpea International genome-wide association panel. **a** Tap root length **b** Lateral root length **c** Number of lateral roots **d** Root diameter **e** Root angle from 1st lateral root **f** Root fresh weight Manhattan plots were created for the genomic regions identified for pooled data of three environments with six models. The displayed MTAs, with peaks showing potential genomic regions linked to root traits. (Models: mrMLM (red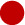), FASTmrMLM (green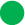), FASTmrEMMA (dark blue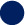), pLARmEB (yellow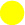), pKWmEB (blue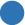) and ISIS.EM.BLASSO (magenta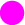))

Genome-wide association mapping was performed using 185 pigeonpea genotypes for RSA traits both for pooled as well as the individual environments (ICRISAT-Patancheru, RARS-Warangal and IIPR-Kanpur). The significant MTAs identified for each RSA trait were given below (Table 3, Fig. 5).

**Table 3:** Identified marker trait associations for root system architectural traits in pigeonpea

Trait	MTAs	Chr	Position	LOD score	− log_10_(*P*)	PVE (%)	Allele	Method	Environment
TRL	CcLG06_21839601	6	21,839,601	15.45	9.68	15.46	GT	1, 4, 6, 7, 8, 9	IIPR, Pooled
TRL	CcLG11_4683590	11	4,683,590	10.44	11.38	7.43	GG	1, 2, 3, 4, 5, 6	IIPR, pooled
TRL	CcLG08_3668059	8	3,668,059	8.38	9.27	6.23	AG	2, 6	Pooled
TRL	CcLG04_2997123	4	2,997,123	10.70	11.65	4.85	AA	2, 6	Pooled
TRL	CcLG07_8576366	7	8,576,366	10.11	11.05	18.73	AG	2, 4, 5, 6	Pooled
TRL	CcLG04_5972718	4	5,972,718	10.88	6.94	12.40	CC	1, 2, 3, 4, 5, 6, 7, 8, 9	IIPR, ICRISAT
LRL	CcLG02_17573019	2	17,573,019	7.86	9.35	12.01	CC	1, 2, 3, 6, 8	IIPR, Pooled
LRL	CcLG01_17476096	1	17,476,096	7.49	8.37	10.09	TT	2, 5	IIPR, Pooled
LRL	CcLG10_14793616	10	14,793,616	8.05	8.94	10.03	CC	1, 2, 4, 6	pooled, IIPR
LRL	CcLG04_12104634	4	12,104,634	6.53	7.74	4.81	GG	1, 2, 3, 5	IIPR, Pooled
LRL	CcLG02_18335854	2	18,335,854	6.33	6.89	7.47	AA	1, 2, 6	ICRISAT, pooled
LRL	CcLG02_21874790	2	21,874,790	8.83	9.74	10.46	AG	1, 2, 5, 6	Pooled
LRL	CcLG11_38239829	11	38,239,829	8.50	9.40	5.87	GG	2,6	Pooled
LRL	CcLG06_14902152	6	14,902,152	5.37	6.18	4.89	GG	2, 3, 4, 7	ICRISAT, IIPR, RARS
LRL	CcLG03_1613404	3	1,613,404	6.05	6.87	7.91	TT	1, 3, 5, 7	ICRISAT, IIPR
LRL	CcLG01_17476721	1	17,476,721	13.23	14.22	10.65	NN	1, 4, 6, 8	ICRISAT, IIPR
LRL	CcLG06_8242342	6	8,242,342	8.23	9.12	13.93	NN	1, 3, 4, 5, 6, 7, 8, 9	IIPR, ICRISAT
LRL	CcLG02_36257342	2	36,257,342	6.71	6.42	4.73	AA	2, 3, 4, 5, 6, 8	IIPR, RARS
NLR	CcLG11_19662589	11	19,662,589	12.14	13.11	8.02	AA	2, 3, 4, 5, 6, 9	ICRISAT, IIPR, Pooled
NRL	CcLG03_20602085	3	20,602,085	9.04	10.75	6.60	GG	1, 2, 3, 4, 5, 6	ICRISAT, RARS, Pooled
NLR	CcLG02_34607221	2	34,607,221	9.77	9.23	14.03	TT	1, 2, 3, 4, 5, 6	RARS, Pooled
NLR	CcLG03_20581935	3	20,581,935	11.54	12.50	3.16	AA	1, 4	Pooled, IIPR
NLR	CcLG07_12804270	7	12,804,270	8.13	9.02	6.46	CC	1, 2, 4, 5, 6	Pooled, ICRISAT
NLR	CcLG11_28734375	11	28,734,375	14.52	10.59	6.19	GG	1, 2, 4	Pooled
NLR	CcLG02_28533358	2	28,533,358	6.57	7.41	4.00	CC	1, 2, 4	Pooled
NLR	CcLG01_4230162	1	4,230,162	10.25	11.19	5.25	TT	2, 4	Pooled
NLR	CcLG01_16026261	1	16,026,261	10.07	8.85	3.03	AA	1, 4	Pooled
NLR	CcLG11_7595283	11	7,595,283	8.12	8.32	8.22	AA	1, 3, 4, 5, 6	ICRISAT, RARS
NLR	CcLG11_36332334	11	36,332,334	6.04	6.87	4.99	TT	1, 3, 4, 6	ICRISAT, RARS
SD	CcLG06_21336203	6	21,336,203	25.21	26.33	1.68	GG	1, 3, 4, 5, 6	ICRISAT, IIPR
RD	CcLG02_22484596	2	22,484,596	6.79	7.64	10.28	AA	1, 2, 4	Pooled
RD	CcLG09_6531175	9	6,531,175	6.84	9.14	9.89	GG	1, 2, 4, 5	Pooled
RD	CcLG09_3522548	9	3,522,548	6.79	9.15	21.57	CC	1, 2, 4	Pooled
RA1	CcLG11_34089457	11	34,089,457	12.31	9.22	11.34	AA	1, 2, 4, 5, 6	ICRISAT, IIPR, RARS, pooled
RA1	CcLG03_16109802	3	16,109,802	9.88	7.03	6.27	AA	2, 4	ICRISAT, IIPR, RARS, pooled
RA1	CcLG01_2584353	1	2,584,353	9.64	7.18	7.62	AA	5, 6	ICRISAT, pooled
RA1	CcLG10_21708404	10	21,708,404	7.85	7.36	5.76	AA	2, 6	RARS, Pooled
RA1	CcLG03_16125624	3	16,125,624	7.85	6.74	17.82	AA	5, 6	Pooled
RA1	CcLG03_16109773	3	16,109,773	7.48	8.35	8.37	TT	6	IIPR, RARS
RA2	CcLG02_7827305	2	7,827,305	8.51	8.80	7.74	CC	3, 4, 5	ICRISAT, Pooled
RA2	CcLG06_16255646	6	16,255,646	7.21	6.42	14.12	CC	1, 3, 4, 5, 6	ICRISAT, Pooled
RFW	CcLG10_19933799	10	19,933,799	9.00	8.82	8.64	TT	1, 2, 4, 4, 6	RARS, Pooled
RFW	CcLG08_16101471	8	16,101,471	10.37	11.31	5.52	CT	1, 4	Pooled
RFW	CcLG10_7777322	10	7,777,322	8.72	9.62	7.90	TT	4, 5	Pooled
RFW	CcLG04_11882078	4	11,882,078	8.59	9.49	5.54	TT	1, 2	Pooled

TRL: Tap root length; LRL: Lateral root length; NLR: Number of lateral roots; SD: Stem Diameter; RD: Root diameter; RA1; root angle from 1st lateral root; RA: Root angle from 2nd lateral root; RFW: Root fresh weight; Chr: Chromosome; LOD: Logarithm of odds; PVE; Phenotypic variation explained; Methods 1–9 represent six mrMLM and 3 GAPIT models; mrMLM, FASTmrMLM, FASTmrEMMA, pLARmEB, pKWmEB, ISISEM-BLASSO, Blink, FarmCPU and Super, respectively

#### Tap root length

Genome-wide association mapping identified a total of six significant MTAs linked to TRL, which were detected in two or more models and confirmed in both pooled data and in more than one environment. These MTAs were distributed across five different chromosomes: 4, 6, 7, 8 and 11. LOD scores, − log_10_(*p*) and PVE value for these MTAs ranged from 8.37 (*CcLG08_3668059*) to 15.449 (*CcLG08_21839601*), 6.94 (CcLG04_5972718) to 11.65 (*CcLG04_2997123*) and 4.84% (*CcLG04_2997123*) to 18.73% (*CcLG07_8576366*), respectively. However, among six MTAs for TRL, *CcLG06_21839601* was identified as the best MTA, as it showed the highest LOD score of 15.45, − log_10_(*p*) value 9.38 and explaining 15.45% of the PVE consistently across four mrMLM (1, 4, 6, 7) and a GAPIT model (8 and 9) in pooled as well as IIPR-Kanpur environment.

#### Lateral root length

Around 12 MTAs are significantly associated with LRL which are distributed on chromosomes 1, 2, 3, 4, 6, 10 and 11. LOD score, − log_10_(*p*) and PVE value of these MTAs vary from 5.37 (*CcLG06_14902152*) to 13.22 (*CcLG01_14476721*); 6.18 (*CcLG06_14902152*) to 14.22 (*CcLG01_14476721*); and 4.89% (*CcLG06_14902152*) to 13.92% (*CcLG06_8242342*), respectively. The MTA *CcLG06_8242342* was found to be highly significant and identified across all models of mrMLM and GAPIT at IIPR-Kanpur and ICRISAT-Patancheru environment.

#### Stem diameter

The MTA *CcLG08_16101471* associated with stem diameter showed LOD score 25.20 and − log_10_(*p*) value of 26.33. It was observed in five mrMLM models at ICRISAT-Patancheru and IIPR-Kanpur environments.

#### Root diameter

In case of root diameter, around three MTAs were found on chromosomes 2 and 9 which are significantly associated with RD. LOD score and − log_10_(*p*) value of these MTAs ranged from 6.78 (*CcLG02_22484596*) to 6.83 (*CcLG09_6531175*) and 7.64 (*CcLG02_22484596*) to 9.15 (*CcLG09_3522548*), respectively. MTA *CcLG09_3522548* showed highest PVE value of 21.56% in pooled data.

#### Number of lateral roots

A total of 11 MTAs was significantly associated with NLR and detected on chromosomes 1, 2, 3, 7 and 11. LOD score and − log_10_(*p*) value of MTAs ranged from 6.03 (*CcLG11_36332334*) to 14.52 (*CcLG11_28734375*) and 6.84 (*CcLG11_36332334*) to 12.56 (*CcLG03_20581935*), respectively. MTA explained the maximum PVE of 14.03% (*CcLG02_34607221*). However, MTA *CcLG11_19662589* was observed in all the mrMLM models with maximum LOD score of 9.04 and − log_10_(*p*) value of 13.11.

For RFW, four significant MTAs were identified on chromosomes 4, 8 and 10, with − log_10_(*p*) values ranging from 8.82 to 11.31. Six MTAs associated with RA1 were detected on chromosomes 1, 3, 10 and 11, explaining 5.76–17.81% PVE among which *CcLG11_34089457* and *CcLG03_16109802* were consistently identified across all environments. In the case of RA2, two significant MTAs were observed on chromosomes 2 and 6, with − log_10_(*p*) values of 6.42 and 8.80, and PVE of 7.73 and 14.12% (Table 3; Fig. 5).

### Allelic distribution of identified MTAs and candidate gene identification

**Fig. 6:**
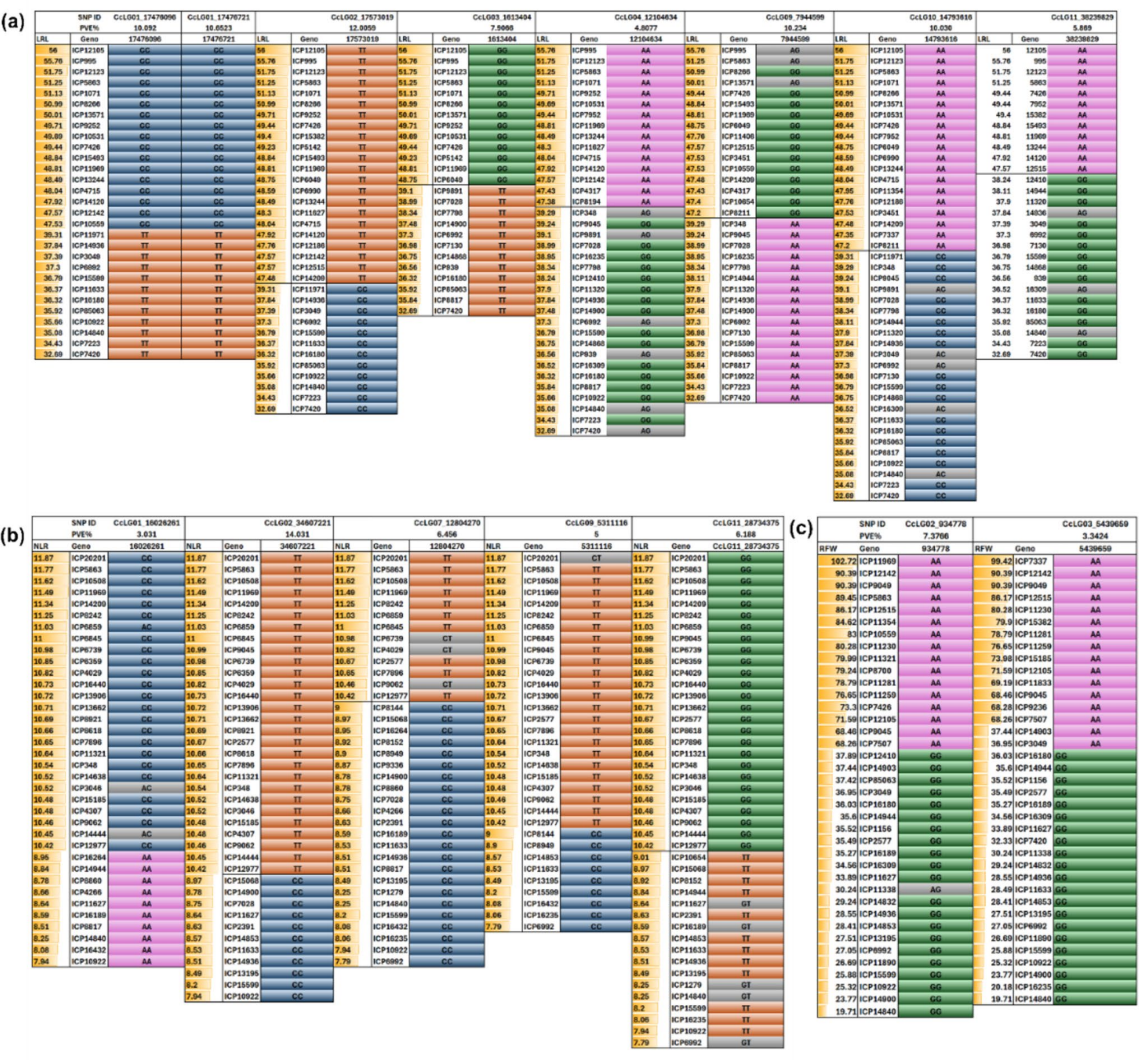
Allelic distribution of significantly associated MTAs among representative PI-GAP genotypes with minimum and maximum trait values, showing the separation of favourable and unfavourable alleles for **a** lateral root length (LRL), **b** number of lateral roots (NLR) and **c** root fresh weight (RFW)

Based on the phenotyping data, a PI-GAP panel genotypes with varied RSA trait performance were selected to assess the efficacy of the identified MTAs to distinguish between the trait values observed in the GWAS population. Allele calls of the identified MTAs for TRL, LRL, NLR, SD, RD and RFW from the selected genotypes were used for assessing the allelic distribution using ‘Axiom_*cajauns* 56 K SNP array’ genotyping data. There was clear differentiation between the favourable and unfavourable, allele present in the associated markers for the selected contrasting genotypes for each RSA traits (Figs. 6 and S4.).

The identified significant MTAs for all eight RSA traits were further used for candidate gene identification using the pigeonpea reference genome version 1. Furthermore, the gene ontology and enrichment analysis were carried to understand the molecular, cellular and biological functions of the identified genes. A total of 14 candidate genes were identified as potentially influencing root characteristics (Table 4), among which 4 candidate genes were putatively associated with the trait TRL. These include: gene *C. cajan_07827* which encodes for the *unc-50 like protein* which was mapped on MTA *CCLG02_35174840*. The gene *C.cajan*_00496 codes for a *LOB domain-containing 22 protein* identified near the MTA *CCLG11_4683590. AT-hook motif nuclear-localized protein 15–29 protein encoding gene C.cajan_13135* associated with MTA *CCLG06_21839601.* Similarly, gene *C.cajan_21512* which encodes for a *root cap* protein family identified on CcLG04_5972718.

**Table 4:** Candidate genes for root architectural traits within ± 100 kb of significant markers

Trait	SNP ID	Gene ID	Orthologous gene IDs	Protein family	GO biological process	GO cellular component	GO molecular function
TRL	CcLG06_21839601	*C.cajan_13135*	*Glyma.11G028800, Vigun02g170300, Phvul.002G151600, AT4G22810*	*AT-hook motif nuclear-localized protein 15–29 (IPR014476)*	vegetative to reproductive phase transition of meristem	nucleus	DNA-binding transcription factor activity, minor groove of adenine–thymine-rich DNA binding
TRL	CcLG04_5972718	*C.cajan_21512*	*Glyma.11G147600, Vigun11g143600, Phvul.011G077800, AT5G60520*	*Root cap (IPR009646)*			
TRL	CcLG02_35174840	*C.cajan_07827*	*Glyma.10G262300, Vigun07g257400, Phvul.007G039900, AT2G15240*	*unc-50 like*	transport	integral component of Golgi membrane	
TRL	CcLG11_4683590	*C.cajan_00496*	*Glyma.07G009500, Vigun10g173000, Phvul.010G138500*	*LOB domain-containing 22*	enhance drought tolerance by regulating key stress-related pathways, including antioxidant activity and root development when overexpressed,		
LRL	CcLG11_38239829	*C.cajan_03474*	*Glyma.12G222300, Vigun05g196200, Phvul.005G083500*	*lanC GCR2*			
LRL	CcLG01_17476096	*C.cajan_20841*	*Glyma.07G061500, Vigun10g139700, Phvul.010G107400, AT2G46240*	*BAG family molecular chaperone regulator 6*			chaperone binding
LRL	CcLG01_17476721	*C.cajan_20841*	*Glyma.07G061500, Vigun10g139700, Phvul.010G107400, AT2G46240*	*BAG family molecular chaperone regulator 6*			chaperone binding
LRL	CcLG02_18335854	*C.cajan_06237*	*Glyma.10G060200, Vigun07g077800, Phvul.007G152500, AT3G53180*	*Protein fluG*	glutamine biosynthetic process		glutamate-ammonia ligase activity, hydrolase activity
LRL	CcLG06_8242342	*C.cajan_11792*		*Protein MAINTENANCE OF MERISTEMS-like (IPR044824)*	meristem maintenance, meristem development		
LRL	CcLG04_12104634	*C.cajan_22050*	*Glyma.13G198900, Vigun11g007100, Phvul.011G208900*	*aspartic protease*	proteolysis		aspartic-type endopeptidase activity, transferase activity
NLR	CcLG11_28734375	*C.cajan_02617*	*Glyma.17G135500, Vigun02g169600, Phvul.002G059900, AT1G12360*	*SNARE-interacting KEULE-like isoform X2*	vesicle docking involved in exocytosis	Role in drought stress	
RD	CcLG09_3522548	*C.cajan_22424*	*Glyma.20G191900, Vigun07g189600, Phvul.007G140800, AT5G04420*	*acyl-binding domain-containing 4-like*			

TRL: Tap root length; LRL: Lateral root length; NLR: Number of lateral roots; SD: Stem Diameter; RD: Root diameter; RA1; root angle from 1st lateral root; RA: Root angle from 2nd lateral root; RFW: Root fresh weight; and GO: Gene ontology

In case of lateral root length (LRL), two significantly associated MTAs, *CcLG01_17476096* and *CCLG01_17476721*, were located near the gene *C. cajan*_20841, which encodes a protein for the *BAG family molecular chaperone regulator 6*. Similarly, gene *C. cajan_06237* which code for *fluG-like protein* was housed at MTA *CcLG02_18335854*. The gene *C.cajan*_22050 encodes for the protein family *Aspartic protease* which identified on MTA *CcLG04_12104634*. In addition, gene *C. cajan_03474*, which encodes a protein belonging to the *LanC-like/GCR2 family,* was identified on *CcLG11_38239829*. Another important gene *C.cajan_11792*, encoding a *MAINTENANCE OF MERISTEMS-like protein*, essential for meristematic cell identity and plant developmental processes was found on MTA *CcLG06_8242342*. For the number of lateral roots, potentially associated gene *C.cajan_02617* codes for *protein SNARE-interacting KEULE-like isoform X2* located on MTA *CcLG11_28734375*.

## Discussion

Pigeonpea has a deeper tap root system, capable of penetrating to a depth of 75 cm and spreading laterally up to 95 cm (Reddy et al*.* 2022). Phenotyping in pots and even cylinders is a challenge, due to lack of natural root growth and establishment. Henceforth, Shovelomics was adopted as a feasible phenotyping method for RSA traits. (Trachsel et al. 2011; Burridge et al. 2020; Bucksch et al. 2014; Colombi et al. 2015; York and Lynch 2015). However, it also has limitations such as loss of tap root tips and partial lateral root loss during excavation as well as post-excavation root wash. Alongside, environmental variability and soil heterogeneity also add on affecting the trait estimation (Li et al. 2022; Tracy 2020; Poorter 2023; Griffin et al. 2025). As this study is the first of its kind in pigeonpea, standardizing the phenotyping protocol was an important step. Accordingly, the physiological maturity stage was identified as the ideal stage for RSA phenotyping, as roots would have attained maximum growth and establishment at this stage. Seedling, vegetative and flowering stages were noticed to have growing roots, while harvesting stage observed root senescence.

The studies in *Phaseolus vulgaris, Phaseolus acutifolius, Glycine max, Vigna unguiculata and Arachis hypogea* have shown that dimorphic root systems are advantageous for tolerance to drought, heat and salinity, whereas shallow root systems are more effective under waterlogging conditions. In the present study, based on tap root length and lateral root width, root system was classified into three categories: deep root types, spreading types and dimorphic types (Burridge et al. 2020). Deep root types were characterized by taproots extending beyond 30 cm below the soil surface, along with shorter lateral roots that spread up to 50 cm horizontally. In contrast, spreading types exhibit shorter taproots (< 30 cm) but possess longer (> 50 cm) and denser lateral roots. Dimorphic types possess both deeper taproots (> 30 cm) and longer lateral roots (> 50 cm) enabling efficient exploration of both surface and deep soil layers (Figs. 1 and S3). This classification could be a valuable framework for breeding pigeonpea genotypes with tolerance to terminal drought, heat, waterlogging and soil salinity (Kumar et al. 2017; Hingane et al. 2015; Fakir et al. 1998). Furthermore, soil types were found to have a greater influence on expression of RSA traits (Correa et al. 2019; Wang et al. 2022), with the Vertisols soil at ICRISAT-Patancheru and the Alfisols at RARS-Warangal favouring spreading root systems in most of the studied genotypes, whereas the Inceptisols soil type at IIPR-Kanpur promoted deeper tap root system compared to the former. This necessitates the further genotype × environments studies to get in depth understanding of root plasticity.

Combined data from three environments revealed significant variation in RSA among the 200 pigeonpea genotypes. The analysis of variance (ANOVA) detected significant genotypic differences (*P* < 0.0001) for root RSA traits, except for RA2 (Table 1). (Zhao et al. 2025; Tripathi et al. 2021; Vadez et al. 2014; Henry et al. 2011). RSA traits such as LRL, NLR and SD were noted to have higher variability with moderate repeatability, indicating phenotypic selection as effective way of genetic improvement. In contrast, root angles (RA1 and RA2) exhibited high variation but lower repeatability, indicating influence of environment on these traits (Heng et al. 2018; Zhao et al. 2025; Ayalew et al. 2017; Burridge et al*.* 2016). Significant correlation was observed among the traits TRL, LRL and RFW, between SD and RD. However, focusing on NLR, SD, RD traits might have positive effects on root length-related traits in pigeonpea (Burton et al. 2014) (Fig. 3). Kumar et al. (2022) and Farooq et al. (2017) suggested root length as one of the selection criteria for yield improvization in chickpea under moisture stress conditions.

RSA traits such as deeper roots, higher root length density and greater root-to-shoot ratio enhance drought avoidance and water-use efficiency (Heng et al. 2018). Genetic variation in RSA, including root angle and thickness, contributes to drought adaptation, with *DRO1* (*Deeper Rooting 1*) doubling yield under severe stress by promoting deep rooting (Uga et al. 2013). Our study on pigeonpea RSA genetic variability supports these findings and it offers an opportunity to evaluate these traits under contrasting soil moisture regimes to improve drought adaptation. Differences in RSA traits underly variation in soil water capture and drought adaptation strategies. For instance, Polania et al. (2017) evaluated 36 bush common bean genotypes using soil cylinder root phenotyping and field-based shoot measurements, revealing that genotypes with larger, deeper roots achieved higher grain yield under drought through improved water uptake, moderated transpiration and sustained vegetative growth (water-spender strategy), whereas genotypes with smaller, shallower roots maintained higher water-use efficiency (water-saver strategy). Lateral roots also facilitated greater soil nitrogen uptake via fine roots, and thicker roots supported enhanced biological nitrogen fixation. These contrasting RSA patterns highlight the potential to select genotypes with either deep, shallow or dimorphic roots tailored to specific drought environments.

Genotypic diversity estimation and principal component analysis (PCA) were performed on 185 pigeonpea genotypes indicating distribution in four cluster. The trait-associated MTAs were identified using the mrMLM and GAPIT packages of RStudio. Within mrMLM, six models were applied: mrMLM, FASTmrMLM, FASTmrEMMA, pLARmEB, pKWmEB and ISIS EM-BLASSO (Zhang et al. 2020a, b) and FarmCPU, Blink, Super from the GAPIT package (Lipka et al. 2012). A total of 45 MTAs are found associated with RSA traits across two environments and pooled data (Table 3), while environment-specific MTAs are listed in Table S4. The allele calls of the selected MTAs could be categorised into two different categories based on the presence of favourable and unfavourable allele in a genotype indicating differential genetic contributions to trait expression. The genotypes ICP 4715 for TRL, ICP 12105 for LRL, ICP 20201 for the NLR, ICP 9414 for RA1, ICP 8266 for RA2 ICP 11969 for RFW and ICP 12512 for RD could be utilized as valuable donors for the improvement of these specific traits (Bomireddy et al. 2024). Candidate genes near MTAs were explored within ± 58 kb flanking regions using the GFF annotation file of the pigeonpea genome (ICPL 87119) (Varshney et al. 2012).

The genes showed association with TRL, including “*Lateral Organ Boundary (LOB) domain”,* was found to have a major role in auxin signalling, cell division, cell elongation and lateral root formation by promoting asymmetric cell divisions in the pericycle cells of *Arabidopsis thaliana* (Berckmans et al. 2011; Zhang et al. 2020a, b; Nguyen et al. 2025). The “*Root cap*” (*C. cajan*_21512) gene associated with MTA *CcLG04_5972718* was having a role in root cap uptake of water and nutrient absorption in maize (Matsuyama et al. 1999). Similarly, another gene “*AT-hook motif nuclear-localized protein*” (*C. cajan_13135*) identified was found to be associated with the MTA *CcLG06_21839601* and noted to be related to *vegetative to reproductive phase transition of the meristem*, as well as regulating diverse aspects of growth and development in plants, being a member of the AHL family as reported in rice and poplar by Zhao et al. 2014 and Lu et al. 2010.

From the current study, the genes *C. cajan_20841, C. cajan_11792, C. cajan_03474, C. cajan_02617* and *C. cajan_22050* were predicted to be involved in the regulation of RSA traits of pigeonpea. Gene *C.cajan_20841* known as “*BAG family molecular chaperone regulator 6*” was found to be associated with MTAs *CcLG01_17476096* and *CcLG01_17476721* of *BAG* families. It has a prominent role in chaperone regulator protein, regulating plant growth, development, under stress conditions. (Wang et al. 2024, 2020), while gene *C. cajan_11792* known as “*Protein MAINTENANCE OF MERISTEMS-like*” was found associated with MTA *CcLG04_8242342*. This gene has a pivotal role in organization of the root apical meristem (RAM) and the shoot apical meristem (SAM). The gene was also found to linked with *Protein MAINTENANCE OF MERISTEMS (MAIN)* and its homologues, MAIN-like 1/2 (MAIL1/2), in *Arabidopsis thaliana,* and they are required to maintain genome stability and cell division activity in meristematic cells. MAIL1 also played a role in cell differentiation as it acts as an important factor for cell fate determination and maintenance throughout plant development (Uhlken et al. 2014; Wenig et al. 2013). The gene “*LanC GCR2*” associated with MTA *CcLG11_38239829* likely involved in stress tolerance and yield-related traits of pigeonpea (Yasin et al. 2019). Another gene associated with MTA *CcLG04_12104634* was “*Aspartic proteases*” widely distributed within the plant kingdom and was involved in protein degradation during normal plant development, programmed cell death and stress response and stress adaptation (Sebastian et al. 2020; Simoes and Faro 2004; Cruz et al. 2001). Another gene associated with NRL “*SNARE-interacting KEULE-like isoform X2*” was found associated with MTA *CclG11_28734375* which was thought to regulate vesicle trafficking and membrane fusion processes essential for plant growth and development in Arabidopsis observed by Assaad et al. (1996) in Table 4.

The tissue-specific expression of the putatively associated gene from the gene expression atlas of pigeonpea (*C.cajan_11096, C.cajan_21512, C.cajan_13135, C.cajan_22424* and *C.cajan_06237*) showed different level of expression predominantly high in root-related tissues, particularly in the radicle and vegetative-stage roots. These genes exhibited strong upregulation during the seedling and vegetative stages, suggesting their crucial roles in root initiation and development. However, a marked downregulation was observed in the reproductive-stage roots, implying differential transcriptional regulation at later developmental phases. In contrast, genes *C.cajan_20841, C.cajan_20862 C.cajan_11096, C.cajan_09366, C.cajan_22409* and *C.cajan_02617* expressed at variable levels across different tissue such as vegetative, reproductive and senescence root tissues, indicating their constitutive involvement in root function and maintenance throughout plant development. The expression of these genes is dynamically regulated according to developmental phase, possibly reflecting changes in physiological requirements and functional roles over time (Singh et al. 2022; Pazhamala et al. 2017; Saxena et al. 2020) (Table S5, Fig. S5). In accordance with this findings, allele-specific markers need to be developed for MTAs like *CcLG01_17476096* (*− *log_10_(*p*) = 8.37; PVE = 10.09), *CcLG04_5972718* (*− *log_10_(*p*) = 6.94; PVE = 12.40), *CcLG06_8242342* (*− *log_10_(*p*) = 9.12; PVE = 13.93) and CcLG11_4683590 (*− *log_10_(*p*) = 11.38; PVE = 7.43) which could serve as potential markers for efficiently differentiating ideal genotypes. Additionally, these MTAs (PVE > 10%) show potential for developing Kompetitive allele-specific polymerase chain reaction (KASP) assays and genotypes with favourable alleles could be used as donors in marker-assisted breeding. Harnessing the dimorphic root system in pigeonpea is a priority for breeding programmes, as this trait could be utilized to redesigning plant architecture. The novel plant type is anticipated to explore biological nitrogen fixation (BNF), carbon capture and tolerance to terminal drought stress.

## Conclusions

Root system architecture (RSA) is emerging as one of the pivotal traits for breeding in the context of climate change, despite the difficulties associated with phenotyping. Tap root length, lateral root length, number of lateral roots, stem diameter, root diameter, root angle and root weight together form some of the main root system architectural traits. The current study developed a standard operating procedure for shovelomics root phenotyping technique in pigeonpea. Based on the current study, the root systems of pigeonpea were grouped into three categories, namely deep, spreading and dimorphic root system. The genetic variability for the RSA traits in pigeonpea identified that the TRL and LRL are the possible traits to be exploited in further breeding programme. The marker trait association revealed 45 significant MTAs associated with 8 RSA traits in pigeonpea. The candidate gene analysis identified genes such as *BAG family molecular chaperone regulator 6* (*CcLG01_17476096* and *CcLG01_17476721*), *root cap* (*CcLG04_5972718*) and *Protein MAINTENANCE OF MERISTEMS (MAIN)* (*CcLG06_8242342*) have a direct role defining root system architecture could help further in abiotic stress tolerance. The identified MTAs could be validated further to detect potential markers related to RSA traits, eventually permitting marker-assisted breeding.

## Supplementary Information

Below is the link to the electronic supplementary material.

Supplementary file1 (DOCX 4809 KB)

Supplementary file2 (DOCX 112 KB)
