# Supplementary material for: Understanding the genetics of root system architecture in pigeonpea [Cajanus cajan (L.) Millsp.]
Source: Theor Appl Genet. 2026 Jan 23;139(1):45. doi: 10.1007/s00122-025-05136-y (PMC12830489; doi:10.1007/s00122-025-05136-y)
Supplement: Supplementary file 1 — Supplementary file1 (DOCX 4809 KB) [file 122_2025_5136_MOESM1_ESM.docx]

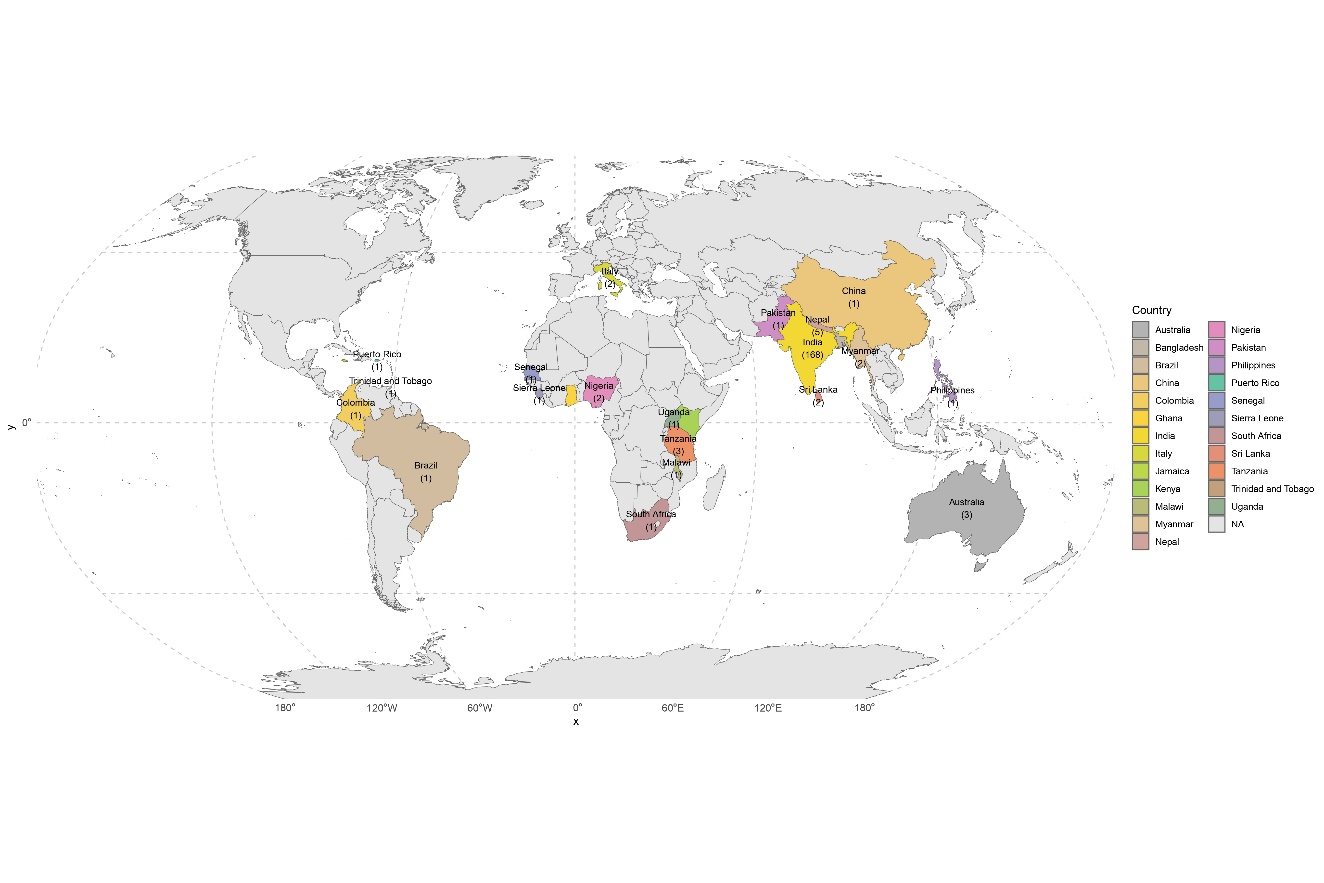


Fig. S1: Geographic Distribution of Pigeonpea International Genome Wide Association Panel (PI-GAP) genotypes of pigeonpea.


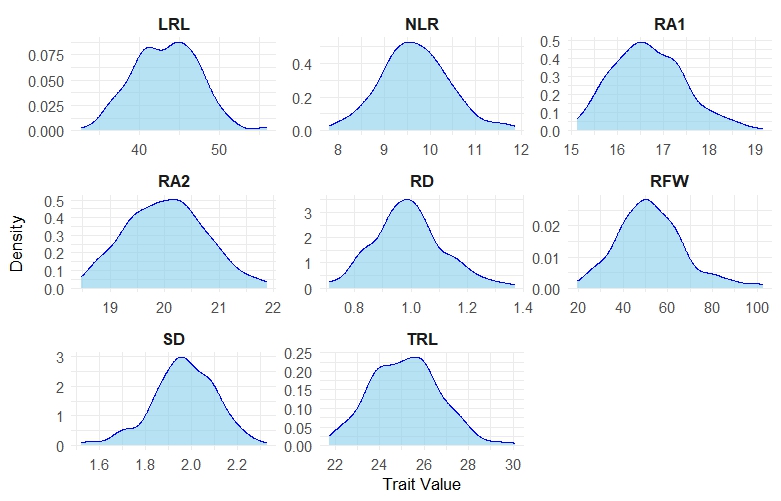


Fig. S2. Density plots showing the distribution of trait mean for eight root system architecture (RSA) traits of pigeonpea genotypes


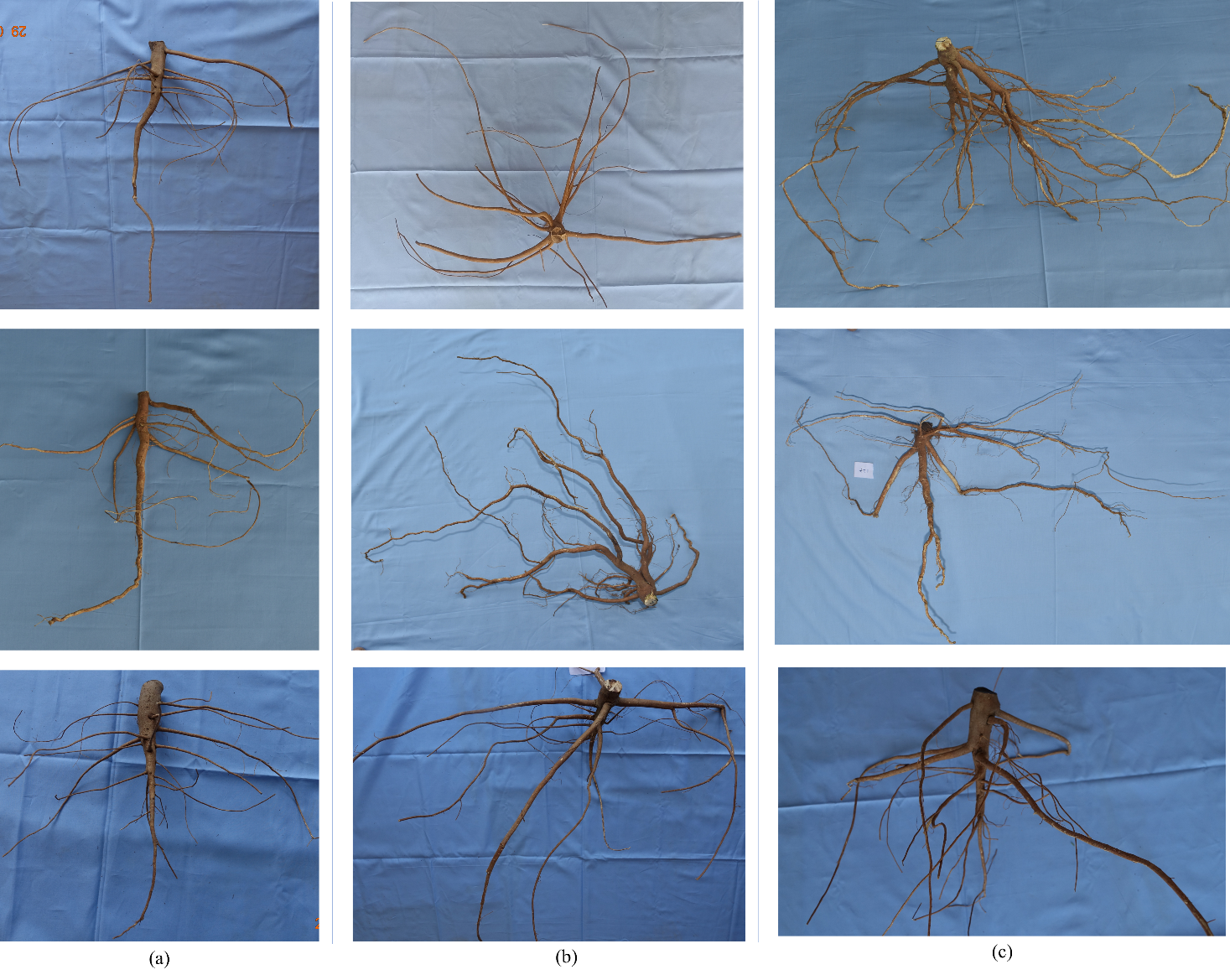


**Figure S3.** Categorization of PI-GAP genotypes into three groups based on root system architecture (RSA) traits: (a) deep root system, (b) spreading root system, and (c) dimorphic root system.

| 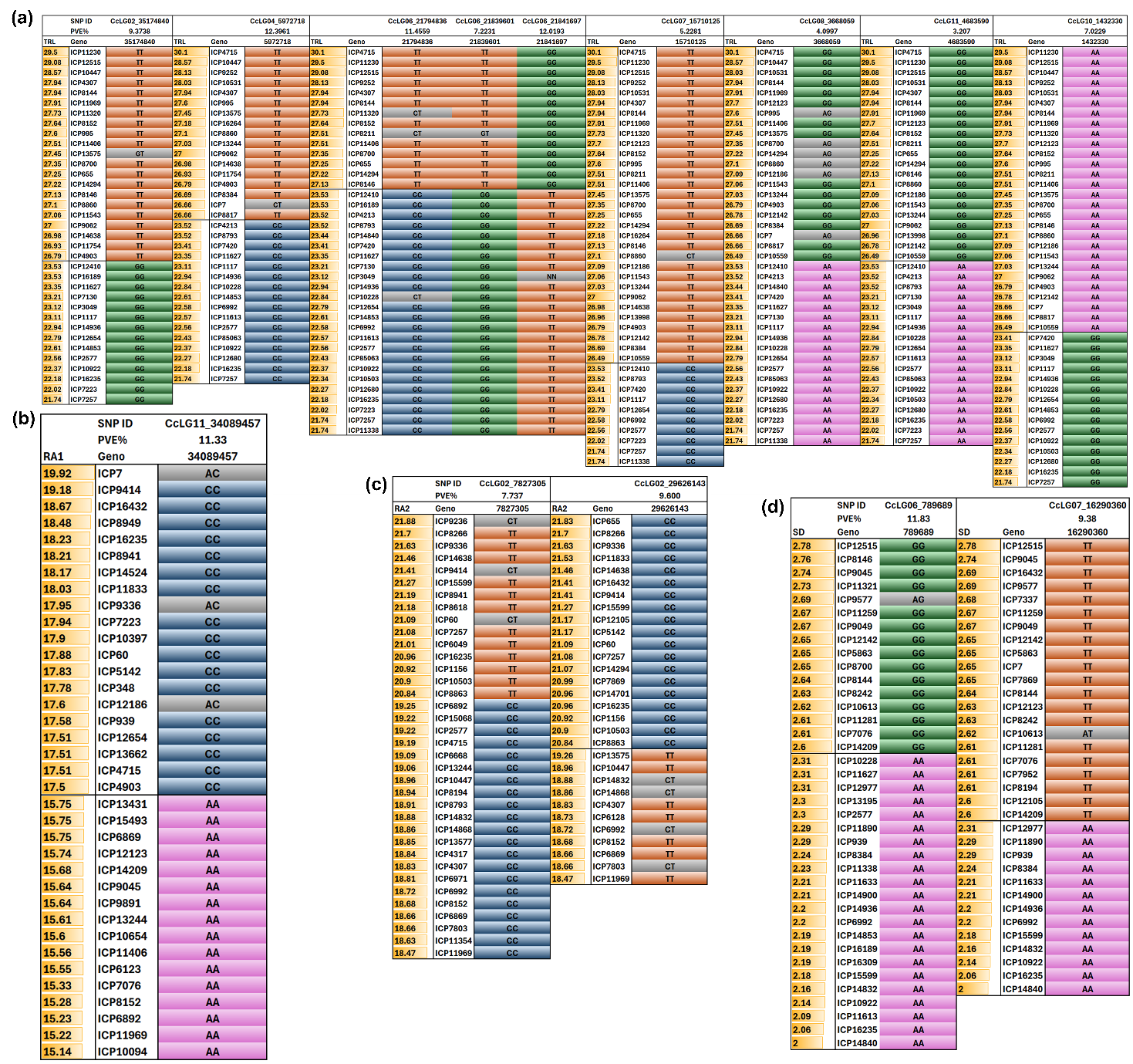 |
| --- |
| Figure S4. Allelic distribution of significantly associated MTAs among representative PI-GAP genotypes with minimum and maximum trait values, showing the separation of favourable and unfavourable alleles for (a) Tap root length (TRL), (b) Root angle from first lateral root (RA1) (c) Root angle from second lateral root (RA2) and (d) Stem dimeter (SD). |


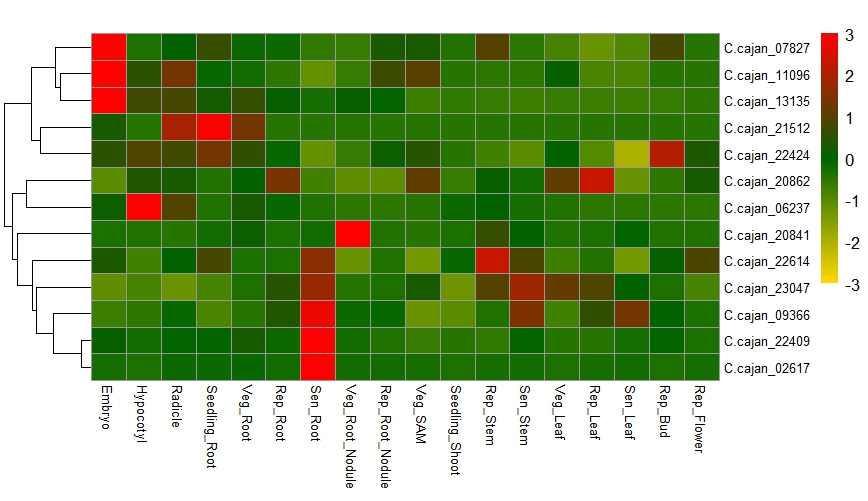


Figure. S5. Tissue-specific expression profiles of identified genes in the pigeonpea reference genome (ICPL 87119 v1). (Samples include: Embryo; Hypocotyl; Radicle; Seedling root; Veg_Root (vegetative root); Rep_Root (reproductive root); Sen_Root (senescence root); Veg_Root_Nodule (vegetative root nodule); Rep_Root_Nodule (reproductive root nodule); Veg_SAM (vegetative shoot apical meristem); Seedling shoot; Rep_Stem (reproductive stem); Sen_Stem (senescence stem); Veg_Leaf (vegetative leaf); Rep_Leaf (reproductive leaf); Sen_Leaf (senescence leaf); Rep_Bud (reproductive bud); and Rep_Flower (reproductive flower).
