# Supplementary material for: Understanding the genetics of root system architecture in pigeonpea [Cajanus cajan (L.) Millsp.]
Source: Theor Appl Genet. 2026 Jan 23;139(1):45. doi: 10.1007/s00122-025-05136-y (PMC12830489; doi:10.1007/s00122-025-05136-y)
Supplement: Supplementary file 2 — Supplementary file2 (DOCX 112 KB) [file 122_2025_5136_MOESM2_ESM.docx]

**Table 1: List of 200 pigeonpea genotypes with their origin, growth habit, MGDI values, and diversity cluster.**

| **Sr. No.** | **Genotype** | **Origin** | **Growth habit** | **MGIDI** | **Diversity Cluster** |
| --- | --- | --- | --- | --- | --- |
| G1 | BDN 2013-1 | India | Semi-spreading | 4.326 |  |
| G2 | BSMR 736 | India | Semi-spreading | 5.954 |  |
| G3 | GRG152 | India | Semi-spreading | 4.914 |  |
| G5 | ICP 10094 | India | Compact | 5.193 | II |
| G6 | ICP 10228 | India | Semi-spreading | 6.79 | I |
| G7 | ICP 10276 | Pakistan | Semi-spreading | 5.512 | I |
| G8 | ICP 10397 | India | Semi-spreading | 4.809 | III |
| G9 | ICP 10447 | India | Semi-spreading | 6.252 | III |
| G10 | ICP 10503 | India | Semi-spreading | 5.749 | III |
| G11 | ICP 10508 | India | Compact | 4.73 | II |
| G12 | ICP 10531 | India | Semi-spreading | 4.314 | IV |
| G13 | ICP 10559 | India | Compact | 3.166 | II |
| G14 | ICP 10613 | India | Semi-spreading | 4.426 | II |
| G15 | ICP 10654 | India | Semi-spreading | 4.795 | II |
| G16 | ICP 1071 | India | Semi-spreading | 5.637 | III |
| G17 | ICP 10922 | Australia | Compact | 7.681 | III |
| G18 | ICP 10963 | India | Semi-spreading | 5.091 | II |
| G19 | ICP 1117 | India | Semi-spreading | 5.671 | I |
| G20 | ICP 11230 | India | Semi-spreading | 2.824 | II |
| G21 | ICP 11259 | India | Compact | 4.736 | II |
| G22 | ICP 1126 | India | Semi-spreading | 5.798 | I |
| G23 | ICP 11281 | India | Compact | 4.658 | II |
| G24 | ICP 11320 | Nepal | Compact | 5.543 | II |
| G25 | ICP 11321 | Nepal | Semi-spreading | 4.83 | II |
| G26 | ICP 11338 | Nepal | Semi-spreading | 6.884 | I |
| G27 | ICP 11354 | Nepal | Semi-spreading | 5.678 | II |
| G28 | ICP 11406 | Nepal | Semi-spreading | 5.68 | II |
| G29 | ICP 11543 | India | Semi-spreading | 4.77 | III |
| G30 | ICP 1156 | India | Semi-spreading | 5.01 | I |
| G31 | ICP 11613 | India | Semi-spreading | 6.669 | III |
| G32 | ICP 11627 | India | Semi-spreading | 5.899 | III |
| G33 | ICP 11633 | India | Semi-spreading | 6.787 | III |
| G34 | ICP 11639 | India | Semi-spreading | 5.336 |  |
| G35 | ICP 11737 | India | Semi-spreading | 6.03 | III |
| G36 | ICP 11754 | India | Semi-spreading | 4.861 | I |
| G37 | ICP 11823 | India | Compact | 4.727 | II |
| G38 | ICP 11833 | India | Compact | 3.161 | II |
| G39 | ICP 11890 | India | Semi-spreading | 6.196 | II |
| G40 | ICP 11969 | India | Compact | 6.027 | II |
| G41 | ICP 11971 | India | Spreading | 6.089 | I |
| G42 | ICP 12105 | Tanzania | Semi-spreading | 3.204 | II |
| G43 | ICP 12123 | Tanzania | Semi-spreading | 5.045 | IV |
| G44 | ICP 12142 | Tanzania | Semi-spreading | 4.661 | II |
| G45 | ICP 12186 | Sri Lanka | Semi-spreading | 3.787 | II |
| G46 | ICP 12410 | India | Semi-spreading | 6.387 | I |
| G47 | ICP 12515 | India | Semi-spreading | 2.959 | II |
| G48 | ICP 12618 | India | Semi-spreading | 5.073 | III |
| G49 | ICP 12654 | India | Semi-spreading | 5.559 | I |
| G50 | ICP 12680 | India | Semi-spreading | 5.609 | I |
| G51 | ICP 1279 | India | Semi-spreading | 5.609 | III |
| G52 | ICP 12977 | India | Semi-spreading | 6.101 | III |
| G53 | ICP 13195 | Australia | Semi-spreading | 6.283 | III |
| G54 | ICP 13244 | Kenya | Semi-spreading | 5.662 | IV |
| G55 | ICP 13304 | Italy | Semi-spreading | 4.645 | IV |
| G56 | ICP 13431 | Malawi | Semi-spreading | 6.207 | IV |
| G57 | ICP 13571 | Nigeria | Semi-spreading | 4.249 | I |
| G58 | ICP 13575 | Sierra Leone | Semi-spreading | 5.161 | IV |
| G59 | ICP 13577 | China | Semi-spreading | 5.252 | IV |
| G60 | ICP 13662 | India | Semi-spreading | 4.56 | III |
| G61 | ICP 13906 | India | Semi-spreading | 5.059 | II |
| G62 | ICP 13998 | South Africa | Compact | 4.77 | II |
| G63 | ICP 14120 | Jamaica | Semi-spreading | 5.452 | IV |
| G64 | ICP 14147 | Brazil | Semi-spreading | 5.449 | IV |
| G65 | ICP 14209 | India | Semi-spreading | 6.018 | II |
| G66 | ICP 14294 | Italy | Compact | 3.587 | II |
| G67 | ICP 14444 | India | Semi-spreading | 5.502 | III |
| G68 | ICP 14524 | India | Semi-spreading | 4.451 | II |
| G69 | ICP 14545 | India | Semi-spreading | 4.815 | II |
| G70 | ICP 14638 | India | Semi-spreading | 4.208 | I |
| G71 | ICP 14701 | India | Compact | 4.224 | IV |
| G72 | ICP 14722 | India | Semi-spreading | 5.194 | I |
| G73 | ICP 14770 | India | Compact | 5.413 | III |
| G74 | ICP 14832 | India | Semi-spreading | 7.769 | III |
| G75 | ICP 14840 | India | Semi-spreading | 7.854 | III |
| G76 | ICP 14853 | India | Semi-spreading | 7.448 | III |
| G77 | ICP 14868 | Philippines | Semi-spreading | 6.525 | III |
| G78 | ICP 14900 | India | Semi-spreading | 6.657 | I |
| G79 | ICP 14903 | India | Semi-spreading | 5.536 | III |
| G80 | ICP 14936 |  |  | 7.123 | III |
| G81 | ICP 14944 | India | Semi-spreading | 6.291 | III |
| G82 | ICP 14951 | India | Compact | 5.825 |  |
| G83 | ICP 15068 | India | Semi-spreading | 6.361 | III |
| G84 | ICP 15185 | India | Semi-spreading | 4.269 | IV |
| G85 | ICP 1535 | India | Semi-spreading | 5.506 | I |
| G86 | ICP 15382 | Nigeria | Semi-spreading | 4.535 | IV |
| G87 | ICP 15493 | Uganda | Semi-spreading | 5.498 | IV |
| G88 | ICP 15599 | India | Compact | 6.584 | III |
| G89 | ICP 16180 | India | Semi-spreading | 6.568 | III |
| G90 | ICP 16189 | India | Semi-spreading | 6.892 | III |
| G91 | ICP 16235 | India | Semi-spreading | 6.654 | III |
| G92 | ICP 16264 | India | Semi-spreading | 5.249 | I |
| G93 | ICP 16309 | India | Semi-spreading | 6.878 | III |
| G94 | ICP 16432 | India | Semi-spreading | 3.394 | II |
| G95 | ICP 16440 | India | Semi-spreading | 5.056 | II |
| G96 | ICP 16674 | India | Semi-spreading | 4.81 | II |
| G97 | ICP 2391 | India | Compact | 4.515 | III |
| G98 | ICP 2405 | India | Semi-spreading | 5.453 | III |
| G99 | ICP 2577 | Myanmar | Semi-spreading | 6.653 | I |
| G100 | ICP 2746 | India | Semi-spreading | 5.8 | III |
| G101 | ICP 3046 | India | Semi-spreading | 5.384 | III |
| G102 | ICP 3049 | India | Semi-spreading | 5.838 | III |
| G103 | ICP 3451 | India | Semi-spreading | 4.929 | III |
| G104 | ICP 348 | India | Semi-spreading | 4.991 | III |
| G105 | ICP 3755 | India | Semi-spreading | 5.538 | II |
| G106 | ICP 4029 | India | Semi-spreading | 5.335 | III |
| G107 | ICP 4213 | India | Semi-spreading | 5.736 | II |
| G108 | ICP 4266 | India | Semi-spreading | 5.642 | II |
| G109 | ICP 4307 | India | Semi-spreading | 5.345 | II |
| G110 | ICP 4317 | India | Semi-spreading | 5.453 | III |
| G111 | ICP 4715 | Ghana | Semi-spreading | 3.884 | I |
| G112 | ICP 4903 | India | Semi-spreading | 3.706 | I |
| G113 | ICP 5142 | India | Semi-spreading | 3.824 | II |
| G114 | ICP 5863 | India | Semi-spreading | 4.137 | II |
| G115 | ICP 60 | India | Semi-spreading | 4.626 | III |
| G116 | ICP 6049 | India | Semi-spreading | 4.237 | I |
| G117 | ICP 6123 | India | Semi-spreading | 5.784 | II |
| G118 | ICP 6128 | Bangladesh | Semi-spreading | 6.665 | I |
| G119 | ICP 6359 | India | Semi-spreading | 5.143 | II |
| G120 | ICP 655 | India | Semi-spreading | 3.489 | III |
| G121 | ICP 6668 | India | Compact | 6.269 | II |
| G122 | ICP 6739 | India | Semi-spreading | 4.217 | IV |
| G123 | ICP 6815 | India | Semi-spreading | 4.855 | II |
| G124 | ICP 6845 | India | Compact | 4.751 |  |
| G125 | ICP 6859 | India | Semi-spreading | 5.301 | II |
| G126 | ICP 6869 | India | Compact | 5.948 | II |
| G127 | ICP 6892 | Puerto Rico | Semi-spreading | 6.28 | IV |
| G128 | ICP 6929 | Trinidad and Tobago | Semi-spreading | 5.174 | IV |
| G129 | ICP 6971 |  |  | 5.887 | I |
| G130 | ICP 6990 | India | Semi-spreading | 5.301 | IV |
| G131 | ICP 6992 | India | Semi-spreading | 7.789 | III |
| G132 | ICP 7 | India | Semi-spreading | 4.038 | I |
| G133 | ICP 7028 | India | Compact | 5.992 | IV |
| G134 | ICP 7035 | India | Semi-spreading | 6.251 |  |
| G135 | ICP 7076 | India | Semi-spreading | 6.068 | II |
| G136 | ICP 7130 | Sri Lanka | Compact | 6.578 | I |
| G137 | ICP 7223 | India | Semi-spreading | 6.259 | I |
| G138 | ICP 7257 | Australia | Compact | 5.532 | I |
| G139 | ICP 7260 | India | Semi-spreading | 5.026 | III |
| G140 | ICP 7266 | Colombia | Semi-spreading | 6.361 | IV |
| G141 | ICP 7269 | Unknown | Compact | 5.421 | III |
| G142 | ICP 7314 | India | Semi-spreading | 6.393 | I |
| G143 | ICP 7337 | India | Compact | 4.841 | IV |
| G144 | ICP 7366 | India | Semi-spreading | 5.106 | III |
| G145 | ICP 7413 | India | Semi-spreading | 5.611 |  |
| G146 | ICP 7420 | India | Semi-spreading | 6.391 | I |
| G147 | ICP 7426 | India | Semi-spreading | 4.541 | II |
| G148 | ICP 7480 | India | Compact | 6.421 | IV |
| G149 | ICP 7487 | India | Semi-spreading | 6.339 |  |
| G150 | ICP 7507 | India | Compact | 4.695 | II |
| G151 | ICP 7798 | India | Semi-spreading | 6.04 | I |
| G152 | ICP 7803 | India | Semi-spreading | 6.732 | I |
| G153 | ICP 7869 | India | Compact | 3.734 | IV |
| G154 | ICP 7896 | India | Compact | 4.445 | IV |
| G155 | ICP 7952 | India | Spreading | 5.653 | I |
| G156 | ICP 8144 | India | Semi-spreading | 3.984 | II |
| G157 | ICP 8146 | India | Semi-spreading | 4.066 | II |
| G158 | ICP 8152 | India | Compact | 5.935 | II |
| G159 | ICP 8194 | Senegal | Semi-spreading | 5.372 | IV |
| G160 | ICP 8211 | India | Spreading | 4.014 | II |
| G161 | ICP 8242 | India | Semi-spreading | 3.974 | II |
| G162 | ICP 8255 | India | Semi-spreading | 5.823 | I |
| G163 | ICP 8266 | India | Semi-spreading | 3.466 | II |
| G164 | ICP 8384 | India | Spreading | 5.302 | III |
| G165 | ICP 8618 | India | Compact | 4.519 | II |
| G166 | ICP 8700 | India | Semi-spreading | 3.551 | II |
| G167 | ICP 8757 | India | Semi-spreading | 5.124 | I |
| G168 | ICP 8776 | India | Semi-spreading | 5.945 |  |
| G169 | ICP 8793 | India | Semi-spreading | 6.631 | III |
| G170 | ICP 8817 | India | Semi-spreading | 5.74 | I |
| G171 | ICP 8860 | India | Compact | 5.114 | I |
| G172 | ICP 8863 | India | Semi-spreading | 4.629 | I |
| G173 | ICP 8921 | India | Semi-spreading | 6.423 | I |
| G174 | ICP 8941 | India | Semi-spreading | 4.209 | I |
| G175 | ICP 8949 | India | Semi-spreading | 4.263 | I |
| G176 | ICP 9045 | India | Semi-spreading | 5.002 | II |
| G177 | ICP 9049 | India | Semi-spreading | 4.128 | II |
| G4 | ICP 9062 | India | Semi-spreading | 4.243 |  |
| G178 | ICP 9236 | India | Semi-spreading | 4.464 | I |
| G179 | ICP 9252 | Myanmar | Compact | 5.227 | II |
| G180 | ICP 9336 | India | Semi-spreading | 5.35 | III |
| G181 | ICP 939 | India | Semi-spreading | 5.645 | I |
| G182 | ICP 9414 | India | Semi-spreading | 4.465 | III |
| G183 | ICP 9577 | India | Compact | 4.349 | II |
| G184 | ICP 9691 | India | Semi-spreading | 4.605 | III |
| G185 | ICP 9750 | India | Semi-spreading | 5.114 | IV |
| G186 | ICP 9891 | India | Semi-spreading | 5.764 | II |
| G187 | ICP 995 | India | Semi-spreading | 4.784 | III |
| G188 | ICPL 19039 | India | Semi-spreading | 6.529 |  |
| G189 | ICPL 19064 | India | Semi-spreading | 4.65 |  |
| G190 | ICPL 20092 | India | Semi-spreading | 6.5 |  |
| G191 | ICPL 20201 | India | Semi-spreading | 5.151 | II |
| G192 | ICPL 20202 | India | Semi-spreading | 4.848 |  |
| G193 | ICPL 20203 | India | Semi-spreading | 5.001 |  |
| G194 | ICPL 20205 | India | Semi-spreading | 6.224 |  |
| G195 | ICPL 85063 | India | Semi-spreading | 6.857 | I |
| G196 | ICPL 87 | India | Semi-spreading | 6.062 |  |
| G197 | ICPL 87119 | India | Semi-spreading | 5.257 |  |
| G198 | ICPL 92016 | India | Semi-spreading | 6.532 |  |
| G199 | ICPL 99050 | India | Semi-spreading | 4.962 |  |
| G200 | TS3R | India | Semi-spreading | 4.49 |  |

Note: G: Genotype number; MGIDI: Multi-Trait Genotype-Ideotype Distance Index

**Table S2:** **Weather parameters of all the trial locations during the 2023 rainy season**

| **Crop season** | **ICRISAT** | | **RARS** | | **IIPR** | | **ICRISAT** | **RARS** | **IIPR** | **ICRISAT** | **RARS** | **IIPR** |
| --- | --- | --- | --- | --- | --- | --- | --- | --- | --- | --- | --- | --- |
| Rainy-2023 | Max Temp | Min Temp | Max Temp | Min Temp | Max Temp | Min Temp | Rainfall (mm) | | | Relative humidity (RH) | | |
| Jun-23 | 36.74 | 24.36 | 38.50 | 26.50 | 27.73 | 38.98 | 140.40 | 9.00 | 298.50 | 70.73 | 63.10 | 63.73 |
| Jul-23 | 29.87 | 22.44 | 30.20 | 23.50 | 26.97 | 35.55 | 456.20 | 758.60 | 172.00 | 87.96 | 88.40 | 85.65 |
| Aug-23 | 31.32 | 22.34 | 31.90 | 24.60 | 26.26 | 35.00 | 47.60 | 39.40 | 171.30 | 87.09 | 85.20 | 83.11 |
| Sep-23 | 30.57 | 22.11 | 33.00 | 23.90 | 26.64 | 35.55 | 255.99 | 138.40 | 139.00 | 90.96 | 91.30 | 84.79 |
| Oct-23 | 32.47 | 18.01 | 32.80 | 21.30 | 20.27 | 33.85 | 0.00 | 5.00 | 26.50 | 90.96 | 87.40 | 81.84 |
| Nov-23 | 30.47 | 18.67 | 31.20 | 20.80 | 14.98 | 30.44 | 19.79 | 2.00 | 0.00 | 89.63 | 89.40 | 90.38 |
| Dec-23 | 28.70 | 14.15 | 28.60 | 16.10 | 11.48 | 23.50 | 3.20 | 2.40 | 15.30 | 90.38 | 90.50 | 93.89 |
| Jan-24 | 30.12 | 16.67 | 30.00 | 17.90 | 7.82 | 17.77 | 0.00 | 0.00 | 8.20 | 86.96 | 90.70 | 91.73 |
| Feb-24 | 33.54 | 18.07 | 32.50 | 19.40 | 10.81 | 26.09 | 0.00 | 0.00 | 28.30 | 82.37 | 87.80 | 82.16 |

Note: Max Temp: Maximum Temperature; Min Temp.: Minimum Temperature; ICRISAT- International Crops Research Institute for the Semi-Arid Tropics; RARS: Regional Agricultural Research Station, Warangal; IIPR: Indian Institute of Pulse Research, Kanpur.

**Table S3. Comparative soil profiles of the three experimental locations.**

| **Sl. No.** | **Sample particular** | **Soil type** | **pH** | **EC** | **OC** | **Avail-P** | **Exch-K** |
| --- | --- | --- | --- | --- | --- | --- | --- |
|  | **Locations** |  |  | **dS/m** | **%** | **ppm** | **ppm** |
| **1** | **ICRISAT (Patancheru)** | Sandy-loam | 7.74 | 0.46 | 0.17 | 12.01 | 178.09 |
| **2** | **RARS (Warangal)** | Vertisols | 7.51 | 0.12 | 0.22 | 12.39 | 42.44 |
| **3** | **IIPR (Kanpur)** | Inseptisols | 7.66 | 0.36 | 0.23 | 21.69 | 39.03 |

**Note:** pH: Potential of Hydrogen; EC: Electrical Conductivity; OC: Organic Carbon; Avail-P: Available Phosphorus; Exch-K: Exchangeable Potassium; ICRISAT- International Crops Research Institute for the Semi-Arid Tropics; Patancheru; RARS: Regional Agricultural Research Station Warangal; IIPR: Indian Institute of Pulse Research, Kanpur.

Table S4. Significant MTAs Identified for Root System Architecture (RSA) Traits from Individual Environments

| **Trait** | **SNPs** | **Chr** | **Position** | **LOD score** | **-log10(P)** | **PVE (%)** | **Genotype** | **Method** | **Locations** |
| --- | --- | --- | --- | --- | --- | --- | --- | --- | --- |
| TRL, SD | CcLG06_21336203 | 6 | 21336203 | 25.205 | 26.33 | 11.04 | GG | 5, 1, 4, 6 | ICRISAT |
| TRL | CcLG06_21841697 | 6 | 21841697 | 10.879 | 11.83 | 12.019 | GG | 2, 3, 8, 9 | IIPR |
| TRL | CcLG07_15710125 | 7 | 15710125 | 7.519 | 8.39 | 5.228 | TT | 6, 7 | IIPR |
| TRL | CcLG10_5860404 | 10 | 5860404 | 8.412 | 9.31 | 8.156 | CC | 6, 4, 1, 7 | IIPR |
| TRL | CcLG02_35174840 | 2 | 35174840 | 8.416 | 9.31 | 9.374 | TT | 6 | RARS |
| TRL | CcLG10_1432330 | 10 | 1432330 | 6.01 | 6.84 | 7.023 | AA | 1, 2 | RARS |
| TRL | CcLG04_5653982 | 4 | 5653982 | 5.482 | 6.29 | 8.496 | CC | 5, 4, 6 | RARS |
| LRL | CcLG04_1693627 | 4 | 1693627 | 7.11 | 7.97 | 5.233 | CC | 4, 6, 2 | ICRISAT |
| LRL | CcLG09_8675747 | 9 | 8675747 | 6.011 | 6.84 | 9.315 | TT | 2, 1, 4 | ICRISAT |
| LRL | CcLG03_13529822 | 3 | 13529822 | 5.239 | 6.04 | 2.877 | AA | 4, 6, 5, 3, 1 | ICRISAT |
| LRL | CcLG08_12492892 | 8 | 12492892 | 7.935 | 8.82 | 9.104 | CC | 6, 5, | IIPR |
| LRL | CcLG01_250390 | 1 | 250390 | 10.548 | 11.49 | 17.445 | CC | 1, 2, 4, 5, 6 | RARS |
| LRL | CcLG02_27487954 | 2 | 27487954 | 6.062 | 6.89 | 9.494 | CC | 1, 7, 8, | RARS |
| LRL | CcLG09_7944599 | 9 | 7944599 | 11.858 | 12.83 | 10.234 | AA | 5, 1, 2, 4 | RARS |
| LRL/TRL | CcLG06_21794836 | 6 | 21794836 |  | 9.74 |  |  | 8 | Pooled |
| LRL | CcLG03_24661099 | 3 | 24661099 |  | 7.03 |  |  | 8 | Pooled |
| LRL | CcLG09_9561198 | 9 | 9561198 |  | 6.7 |  |  | 8 | Pooled |
| NLR | CcLG09_5311116 | 9 | 5311116 | 7.829 | 8.71 | 2.87 | TT | 4 | RARS |
| NLR | CcLG01_17667014 | 1 | 17667014 | 5.673 | 6.49 | 2.21 | CC | 6 | RARS |
| SD | CcLG07_16290360 | 7 | 16290360 | 13.932 | 14.93 | 9.38 | TT | 5, 1 | IIPR |
| SD | CcLG06_789689 | 6 | 789689 | 13.759 | 14.76 | 11.835 | NN | 4, 5, 6, 1 | RARS |
| SD | CcLG10_17601941 | 10 | 17601941 | 12.448 | 13.42 | 22.947 | AA | 1, 4, 6, 5 | RARS |
| SD | CcLG11_32032969 | 11 | 32032969 | 9.847 | 10.78 | 8.459 | AA | 5, 1, 4, 6, 3 | RARS |
| SD | CcLG03_20673246 | 3 | 20673246 | 9.792 | 10.72 | 0.376 | GG | 4, 2 | IIPR |
| SD | CcLG11_35870532 | 11 | 35870532 | 9.512 | 10.44 | 2.363 | CC | 2, 1, 5 | RARS |
| SD | CcLG11_16110937 | 11 | 16110937 | 9.22 | 10.13 | 0.90 | CC | 2 , 4 | IIPR |
| SD | CcLG04_3337191 | 4 | 3337191 | 9.01 | 9.93 | 4.49 | CC | 4, 5, 1 | RARS |
| SD | CcLG06_8059534 | 6 | 8059534 | 7.39 | 8.26 | 3.58 | CC | 2, 5, 1 | RARS |
| RD | CcLG03_11211329 | 3 | 11211329 | 5.22 | 6.02 | 6.14 | GG | 1, 2, 4 | Pooled |
| RD | CcLG11_4188342 | 11 | 4188342 | 3.62 | 6.07 | 2.88 | TT | 1, 2, 4 | Pooled |
| RD | CcLG11_1162886 | 11 | 1162886 | 8.85 | 9.76 | 8.33 | AA | 1,2,4,5 | IIPR |
| RA1 | CcLG11_12121292 | 11 | 12121292 | 8.22 | 9.11 | 13.89 | AG | 1, 6 | ICRISAT |
| RA1 | CcLG06_14114640 | 6 | 14114640 | 7.03 | 7.89 | 5.76 | AA | 4, 2, 6 | ICRISAT |
| RA2 | CcLG06_6356584 | 6 | 6356584 | 12.37 | 13.35 | 7.58 | AA | 5, 3, 4, | RARS |
| RA2 | CcLG02_29626143 | 2 | 29626143 | 10.66 | 11.62 | 9.60 | CC | 6, 1 | RARS |
| RA2/RD | CcLG03_7754954 | 3 | 7754954 | 10.30 | 11.24 | 4.44 | AA | 4, 5 | IIPR |
| RA2 | CcLG08_9266138 | 8 | 9266138 | 9.83 | 10.76 | 8.13 | CC | 5, 4, 6 | ICRISAT |
| RA2/RD | CcLG05_3055104 | 5 | 3055104 | 9.76 | 10.69 | 11.37 | AA | 4, 5 | IIPR |
| RA2 | CcLG07_13833122 | 7 | 13833122 | 8.95 | 9.86 | 9.56 | AA | 5,4, 1 | ICRISAT |
| RA2/RD | CcLG11_15603133 | 11 | 15603133 | 8.67 | 9.57 | 7.49 | AG | 4, 6, 3, 1 | IIPR |
| RA2 | CcLG10_2128859 | 10 | 2128859 | 6.44 | 7.28 | 9.39 | GG | 5, 4, 6, 3 | IIPR |
| RFW | CcLG10_11678556 | 10 | 11678556 | 14.33 | 13.34 | 12.09 | CC | 4, 5, 1, 6, 3 | ICRISAT |
| RFW | CcLG11_33555333 | 11 | 33555333 | 10.85 | 11.80 | 5.73 | CC | 4, 3, 1, 6 | ICRISAT |
| RFW | CcLG07_16009798 | 7 | 16009798 | 9.53 | 10.45 | 10.76 | CC | 4, 1, 6, 2, 5 | RARS |
| RFW | CcLG03_17721022 | 3 | 17721022 | 9.02 | 9.93 | 10.26 | CC | 6, 3, 1, 2, 5 | ICRISAT |
| RFW | CcLG02_7116222 | 2 | 7116222 | 8.13 | 9.02 | 11.55 | CC | 6, 4, | RARS |
| RFW | CcLG03_5439659 | 3 | 5439659 | 8.10 | 8.99 | 3.34 | GG | 4, 2, 1, 3 | ICRISAT |
| RFW | CcLG02_934778 | 2 | 934778 | 7.98 | 8.87 | 7.38 | GG | 4, 5 | IIPR |
| RFW | CcLG09_10238107 | 9 | 10238107 | 7.84 | 8.72 | 13.17 | AA | 4, 1, 3 | ICRISAT |

Note: TRL: Tap root length; LRL: Lateral root length; NLR: Number of lateral roots; SD: Stem Diameter; RD: Root diameter; RA1; root angle from 1st lateral root; RA: Root angle from 2^nd^ lateral root; RFW: Root fresh weight; Chr: Chromosome; LOD: Logorithm of Odds; PVE; Phenotypic Variation Explained; Methods 1–9 represent six mrMLM and 3 GAPIT models; mrMLM, FASTmrMLM, FASTmrEMMA, pLARmEB, pKWmEB, ISISEM-BLASSO, Blink, FarmCPU and Super respectively; ICRISAT- International Crops Research Institute for the Semi-Arid Tropics; Patancheru; RARS: Regional Agricultural Research Station Warangal; IIPR: Indian Institute of Pulse Research, Kanpur.

| **Gene_ID** | **Embryo** | **Hypocotyl** | **Radicle** | **Seedling_Root** | **Veg_Root** | **Rep_Root** | **Sen_Root** | **Veg_Root_Nodule** | **Rep_Root_Nodule** | **Veg_SAM** | **Seedling_Shoot** | **Rep_Stem** | **Sen_Stem** | **Veg_Leaf** | **Rep_Leaf** | **Sen_Leaf** | **Rep_Bud** | **Rep_Flower** |
| --- | --- | --- | --- | --- | --- | --- | --- | --- | --- | --- | --- | --- | --- | --- | --- | --- | --- | --- |
| *C.cajan_20841* | 12.58 | 11.25 | 2.05 | 29.83 | 80.23 | 19.06 | 29.37 | 605.95 | 4.51 | 1.54 | 16.61 | 153.86 | 66.55 | 4.93 | 20.03 | 60.31 | 6.03 | 3.85 |
| *C.cajan_20862* | 4.06 | 29.35 | 26.93 | 15.51 | 22.26 | 46.53 | 9.69 | 3.00 | 2.81 | 41.60 | 11.38 | 23.43 | 18.61 | 41.64 | 61.88 | 0.83 | 13.37 | 26.88 |
| *C.cajan_07827* | 83.49 | 33.45 | 38.11 | 46.89 | 36.26 | 35.83 | 29.81 | 28.71 | 42.17 | 41.54 | 32.81 | 52.04 | 30.83 | 26.53 | 20.72 | 24.84 | 49.28 | 31.59 |
| *C.cajan_06237* | 100.66 | 633.87 | 218.21 | 23.53 | 116.60 | 67.48 | 20.76 | 5.26 | 2.01 | 8.01 | 59.37 | 82.57 | 41.55 | 15.32 | 4.62 | 0.64 | 2.91 | 0.62 |
| *C.cajan_09366* | 12.82 | 13.85 | 16.51 | 11.35 | 14.11 | 19.81 | 34.75 | 16.30 | 16.69 | 9.24 | 10.54 | 14.81 | 26.64 | 12.17 | 20.99 | 25.71 | 17.54 | 14.96 |
| *C.cajan_21512* | 33.33 | 0.52 | 108.82 | 152.10 | 82.12 | 0.74 | 0.26 | 1.21 | 0.71 | 0.00 | 0.79 | 0.95 | 0.00 | 0.00 | 0.18 | 0.07 | 1.73 | 0.31 |
| *C.cajan_11096* | 42.07 | 20.49 | 27.62 | 14.99 | 13.80 | 11.22 | 5.49 | 10.22 | 21.78 | 25.06 | 11.97 | 11.03 | 11.29 | 16.58 | 8.08 | 7.98 | 11.48 | 11.88 |
| *C.cajan_13135* | 31.47 | 10.92 | 11.45 | 7.09 | 10.21 | 5.76 | 3.18 | 6.13 | 4.87 | 0.19 | 0.69 | 0.62 | 0.00 | 0.43 | 0.00 | 0.06 | 0.36 | 1.50 |
| *C.cajan_22409* | 12.13 | 8.40 | 10.71 | 10.15 | 13.97 | 8.75 | 55.17 | 8.14 | 6.05 | 3.80 | 5.65 | 4.58 | 10.20 | 5.37 | 5.64 | 7.59 | 10.34 | 7.31 |
| *C.cajan_22424* | 38.63 | 44.19 | 41.80 | 48.73 | 39.76 | 31.48 | 19.05 | 25.73 | 34.96 | 38.46 | 27.73 | 23.85 | 20.50 | 33.08 | 20.99 | 9.14 | 57.11 | 36.95 |
| *C.cajan_22614* | 29.71 | 22.12 | 27.93 | 33.40 | 25.03 | 25.30 | 38.79 | 18.59 | 24.87 | 17.87 | 26.93 | 44.12 | 33.68 | 22.49 | 24.67 | 17.95 | 27.99 | 33.77 |
| *C.cajan_23047* | 0.82 | 1.23 | 0.61 | 1.23 | 1.97 | 3.10 | 5.05 | 1.76 | 1.92 | 2.86 | 0.51 | 3.86 | 5.15 | 4.13 | 3.78 | 2.48 | 1.97 | 1.21 |
| *C.cajan_02617* | 30.72 | 21.84 | 43.12 | 44.11 | 55.42 | 41.75 | 601.43 | 37.58 | 40.68 | 27.81 | 24.89 | 30.75 | 39.39 | 24.34 | 27.88 | 40.74 | 24.20 | 28.99 |

Table S5: Expression Analysis of Candidate Genes Across Aboveground and Belowground Tissues of Pigeonpea

Note: Different aboveground and belowground tissues include Embryo; Hypocotyl; Radicle; Seedling Root; Vegetative Root (Veg_Root); Reproductive Root (Rep_Root), Senescent Root (Sen_Root); Vegetative Root Nodule (Veg_Root_Nodule); Reproductive Root Nodule (Rep_Root_Nodule); Seedling Shoot; Vegetative Shoot Apical Meristem (Veg_SAM); Reproductive Stem (Rep_Stem); Senescent Stem (Sen_Stem); Vegetative Leaf (Veg_Leaf); Reproductive Leaf (Rep_Leaf); Senescent Leaf (Sen_Leaf); Reproductive Bud (Rep_Bud); Reproductive Flower (Rep_Flower).
